# Development of a consensus core dataset in juvenile dermatomyositis for clinical use to inform research

**DOI:** 10.1136/annrheumdis-2017-212141

**Published:** 2017-10-30

**Authors:** Liza J McCann, Clarissa A Pilkington, Adam M Huber, Angelo Ravelli, Duncan Appelbe, Jamie J Kirkham, Paula R Williamson, Amita Aggarwal, Lisa Christopher-Stine, Tamas Constantin, Brian M Feldman, Ingrid Lundberg, Sue Maillard, Pernille Mathiesen, Ruth Murphy, Lauren M Pachman, Ann M Reed, Lisa G Rider, Annet van Royen-Kerkof, Ricardo Russo, Stefan Spinty, Lucy R Wedderburn, Michael W Beresford

**Affiliations:** 1 Department of Paediatric Rheumatology, Alder Hey Children’s NHS Foundation Trust, Liverpool, UK; 2 Department of Paediatric Rheumatology, Great Ormond Street Hospital for Children NHS Foundation Trust, London, UK; 3 Arthritis Research UK Centre for Adolescent Rheumatology, University College London, University College London Hospital, London, UK; 4 Division of Pediatric Rheumatology, IWK Health Centre and Dalhousie University, Halifax, Nova Scotia, Canada; 5 Division of Rheumatology, Università degli Studi di Genova and Istituto Giannina Gaslini Pediatria II-Reumatologia, Genoa, Italy; 6 Department of Biostatistics, MRC North West Hub for Trials Methodology Research, University of Liverpool, Liverpool, UK; 7 Department of Clinical Immunology, Sanjay Gandhi Postgraduate Institute of Medical Sciences, Lucknow, India; 8 Johns Hopkins Myositis Center, Johns Hopkins University, Baltimore, Maryland, USA; 9 2nd Department of Paediatrics, Semmelweis University, Budapest, Hungary; 10 Department of Pediatrics, The Hospital for Sick Children and University of Toronto, Toronto, Canada; 11 Department of Medicine, Rheumatology Unit, Karolinska Institutet, Karolinska University Hospital, Stockholm, Sweden; 12 Department of Paediatrics and Adolescent Medicine, Naestved Hospital, Region Zeeland, Naestved, Denmark; 13 Department of Dermatology, Royal Hallamshire Hospital, Sheffield, UK; 14 Department of Pediatrics, Feinberg School of Medicine, Northwestern University, Chicago, Illinois, USA; 15 Division of Rheumatology, Ann and Robert H. Lurie Children’s Hospital of Chicago, Chicago, Illinois, USA; 16 Centre for Clinical Immunology, The Stanley Manne Children’s Research Centre, Chicago, Illinois, USA; 17 Department of Paediatrics, Duke University, Durham, North Carolina, USA; 18 Environmental Autoimmunity Group, Clinical Research Branch, National Institute of Environmental Health Sciences, Bethesda, Maryland, USA; 19 Department of Paediatric Immunology and Rheumatology, Wilhelmina Children’s Hospital University Medical Centre, Utrecht, The Netherlands; 20 Department of Paediatric Immunology and Rheumatology, Paediatric Hospital Dr. Juan P. Garrahan, Buenos Aires, Argentina; 21 Department of Paediatric Neurology, Alder Hey Children’s NHS Foundation Trust, Liverpool, UK; 22 Infection, Immunology, and Rheumatology Section, UCL GOS Institute of Child Health, University College London, London, UK; 23 NIHR-Biomedical Research Centre at GOSH, London, UK; 24 Department of Women’s and Children’s Health, Institute of Translational Medicine, University of Liverpool, Liverpool, UK

**Keywords:** dermatomyositis, patient perspective, outcomes research, autoimmune diseases, multidisciplinary team-care

## Abstract

**Objectives:**

This study aimed to develop consensus on an internationally agreed dataset for juvenile dermatomyositis (JDM), designed for clinical use, to enhance collaborative research and allow integration of data between centres.

**Methods:**

A prototype dataset was developed through a formal process that included analysing items within existing databases of patients with idiopathic inflammatory myopathies. This template was used to aid a structured multistage consensus process. Exploiting Delphi methodology, two web-based questionnaires were distributed to healthcare professionals caring for patients with JDM identified through email distribution lists of international paediatric rheumatology and myositis research groups. A separate questionnaire was sent to parents of children with JDM and patients with JDM, identified through established research networks and patient support groups. The results of these parallel processes informed a face-to-face nominal group consensus meeting of international myositis experts, tasked with defining the content of the dataset. This developed dataset was tested in routine clinical practice before review and finalisation.

**Results:**

A dataset containing 123 items was formulated with an accompanying glossary. Demographic and diagnostic data are contained within form A collected at baseline visit only, disease activity measures are included within form B collected at every visit and disease damage items within form C collected at baseline and annual visits thereafter.

**Conclusions:**

Through a robust international process, a consensus dataset for JDM has been formulated that can capture disease activity and damage over time. This dataset can be incorporated into national and international collaborative efforts, including existing clinical research databases.

## Introduction

Juvenile dermatomyositis (JDM) is associated with significant morbidity and mortality.^1–3^ To better understand this rare disease,^4^ international collaboration is essential. This is feasible with the development of national and international electronic web-based registries and biorepositories.^5 6^ For good clinical care and to aid comparison of data between groups, it is crucial to have a common dataset that clinicians and researchers collect in a standardised way, with items clearly defined. The International Myositis and Clinical Studies (IMACS) Group^7–9^ and Paediatric Rheumatology International Trials Organisation (PRINTO)^10–12^ JDM core sets were developed predominantly for research studies. Existing myositis registries include partially overlapping but different dataset items, making comparison between groups challenging.^13^ This study aimed to define optimal items from existing datasets that would be useful to collect in routine practice, within accessible disease-specific registries, that, when measured over time, would help capture disease outcome/treatment response, which would facilitate both patient care and translational research.

## Methods

**Figure 1:**
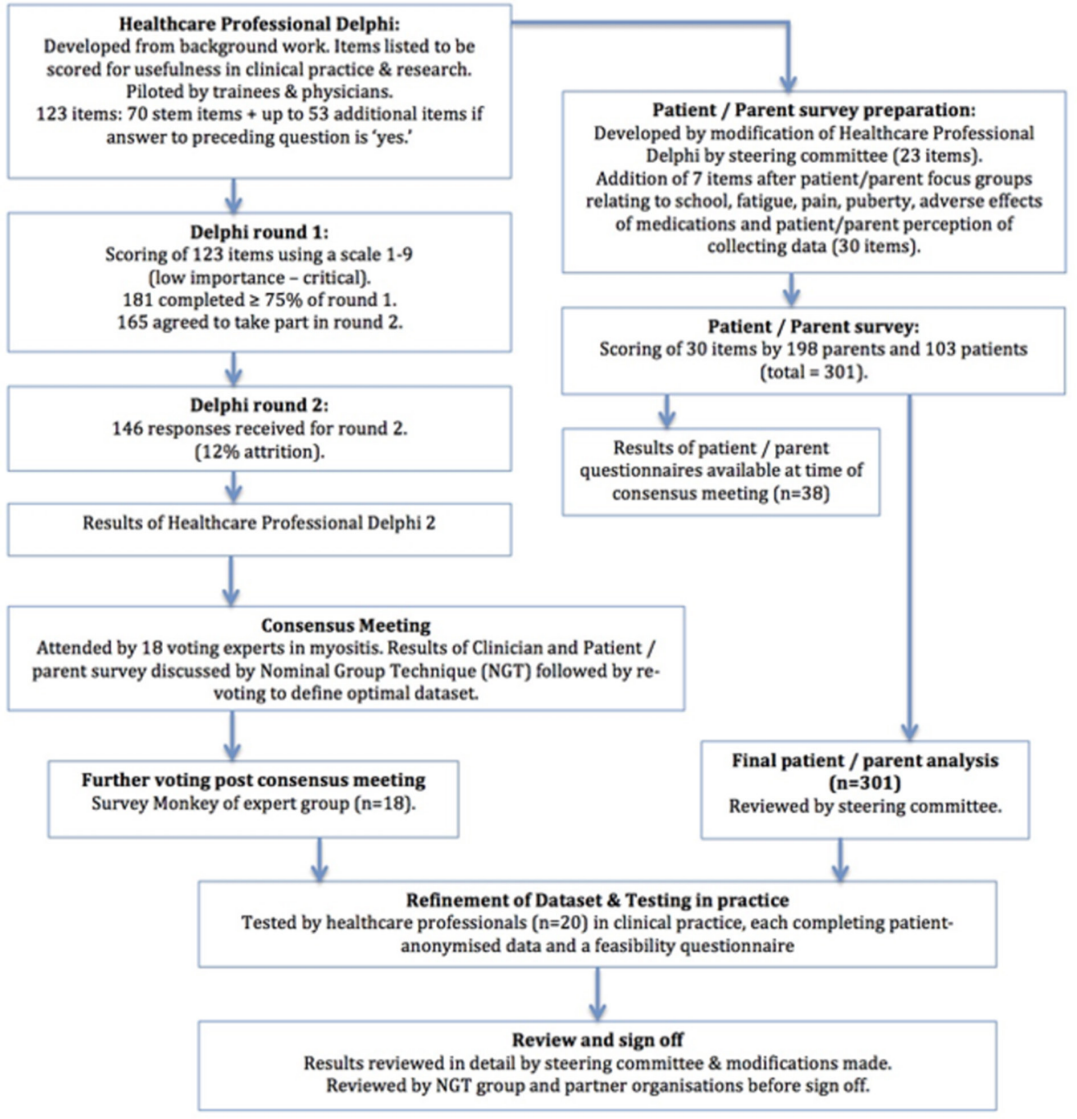
Flow chart showing study overview.

The study protocol and background work have been published.^13 14^ The study is registered on the Core Outcome Measures in Effectiveness Trials initiative database.^15^ The Core Outcome Set—STAndards for Reporting standards for reporting were followed.^16^ The study overview is shown in figure 1.

### Background work

A steering committee (SC) developed a prototype dataset by scrutinising all items within existing international databases of juvenile-onset myositis (JM) and adult-onset myositis,^1 17–19^ informed by a literature search and detailed analysis of the UK Juvenile Dermatomyositis Cohort Biomarker Study and Repository (JDCBS).^13 19^ Leading representatives of each partner organisation^9 12 17 20 21^ detailed in the study protocol^14^ approved the template/provisional dataset.

### Stakeholder groups

This study design aimed to employ representation from healthcare professionals with experience in myositis working as physicians, allied health professionals or clinical scientists in paediatric or adult medicine within rheumatology, neurology or dermatology^14^ and consumers (patients with JM and their parents or carers).

### Healthcare professional Delphi process

A two-stage Delphi process was undertaken.^14^ Items contained within the prototype dataset were listed and further modified by the SC to ensure clarity. The items were formatted into a custom-made electronic questionnaire, piloted before distribution. After modifications, the Delphi template included 70 items with an additional 53 conditional on previous response (detailed in online supplementary table S1). Participation was invited via membership lists of IMACS, Childhood Arthritis and Rheumatology Research Alliance (CARRA), Juvenile Dermatomyositis Research Group (JDRG) UK and Ireland, Paediatric Rheumatology European Society (PReS) JDM working party and PRINTO Centre Directors. These are representative of international paediatric rheumatology and myositis specialty groups, capturing opinion of clinicians, scientists and allied health professionals. The estimated membership of these groups totals more than 1000. However, the majority of members belong to more than one organisation and membership lists include retired/non-active members or specialists working in adult-onset myositis potentially less inclined to answer a paediatric-specific survey.^14^ Participants were asked to rate the importance of each item for clinical practice and separately for value in research, using a scale of 1–9: 1–3 (of low importance), 4–6 (important but not critical) and 7–9 (critical).^14^ An option of ‘unable to score’ was given and free text comments were allowed. Delphi 2 was sent to participants who scored 75% or more of the items in round 1 of the Delphi. Each participant was asked to re-score each item, having been shown the distribution of scores for the group as a whole and their own score.

10.1136/annrheumdis-2017-212141.supp1 Supplementary file 1

### Patient and parent survey

The healthcare professionals’ survey was modified into separate parent and patient questionnaires as per protocol,^14^ formatted for computer or paper format completion. The questionnaires and age-appropriate information leaflets were reviewed by patient and public involvement coordinators and by parent/young people’s focus groups.^14^ The focus groups also reviewed patient/parent-reported outcome measures (PROMs) used for JDM and other rheumatology conditions,^22–27^ and opinions were summarised (online supplementary table S2). Thirty items were included in patient/parent questionnaires; 23 from adaptation of the Delphi (combining or simplifying items from the healthcare professional questionnaire and selecting items particularly relevant to patients/parents), 2 additional questions added by the SC to determine patient/parent perspectives on collecting and storing information, plus 5 questions suggested by patients/parents within focus groups (online supplementary table S1). The scoring system was simplified into three categories of ‘not that important’, ‘important’ and ‘really important’. An option of ‘unable to score’ was given and free text comments were allowed. Participation was open to any patient with JM—child/adult, or any parent/carer of a child with JM. Patients with adult-onset myositis (onset ≥18 years) were excluded. Information leaflets and questionnaires were in English only; translators could be used if available. Patients/parents were signposted to the study via email distribution lists/websites of North American and UK patient support groups (Cure JM and Myositis UK),^28 29^ the lead of the JDRG patient/parent groups and JDRG coordinator.^20^ In addition, following site-specific ethics approval, UK centres participating in the JDCBS^19 30^ and a Netherlands site invited patients/parents to participate.

### Data analysis

For each item, the number and percentage of participants who scored the item and the distribution of scores (grades 1–9) were summarised for each stakeholder group. Consensus definitions were applied as ‘consensus in’ versus ‘equivocal’ or ‘consensus out’ according to predefined consensus definitions (table 1).

**Table 1:** Definition of consensus for each stage of the study (defined a priori)

Consensus classification	Description	Definition of consensus		
		Healthcare professionals’ Delphi	Patient/parent survey	Consensus meeting*
Consensus in	Consensus that outcome should be included in core set	≥70% of participants scoring ‘7–9’ ‘critical for decision-making’	≥70% of participants scoring ‘really important’	≥80% of participants voting for inclusion in core outcome set
Consensus out	Consensus that outcome should not be included in the core outcome set	≥70% of participants scoring ‘1–3’ ‘low importance’	≥70% of participants scoring ‘not that important’	<80% of participants voting for inclusion in core outcome set
Equivocal	Uncertainty about importance of outcome	All other responses	All other responses	Further discussion by NGT and re-voting allowed

*More stringent consensus cut-off for consensus meeting.

NGT, nominal group technique.

### Consensus meeting

Eighteen voting delegates were invited to a 2-day consensus meeting, led by a non-voting facilitator (MWB). International representatives were experts in myositis from paediatric rheumatology/myositis groups and professionals who care for patients with myositis including neurologists, dermatologists, adult rheumatologists and physiotherapists. Prior to the meeting, delegates were sent a summary of results to review. During the consensus meeting, Delphi 2 results and patient/parent results were presented for each item—as shown in online supplementary figure 1. Items achieving ‘consensus in’ within the Delphi and patient/parent questionnaires were voted on immediately. Those not achieving ‘consensus in’ were discussed by nominal group technique. Consensus was defined a priori as ≥80% (table 1). Discussion and re-voting allowed refinement of items or associated definitions. The process continued until consensus was reached or until it was clear that consensus would not be reached.

10.1136/annrheumdis-2017-212141.supp2 Supplementary file 2

### Testing in practice

The proposed dataset was formatted into three sections (forms A, B and C) and tested in clinical practice. Members of the expert group were asked to test the dataset themselves and/or delegate a member of their department unfamiliar with the dataset. Clinicians completed patient-anonymised data on one to two patients under their care and a feasibility questionnaire (online supplementary table S3). Feedback was considered by the SC and refinements made. The dataset was sent to the expert group, including representatives of partner organisations (IMACS, CARRA, PRINTO, PReS JDM working group, JDRG, Euromyositis) for comment.

## Results

Two hundred and sixty-two healthcare professionals accessed the system (26% of the estimated total membership of specialty groups). 181/262 (69%) completed ≥75% of Delphi 1 (June–September 2014). One hundred and sixty-five agreed to take part in Delphi 2 (November 2014–January 2015); from these, 146 replies were received (12% attrition). One hundred and seventy-two participants provided full demographic data in round 1 showing that survey responses were received from Europe (44%), North America (34%), Latin America (12%), Asia (6%), Australia/Oceania (0.5%), Middle East (3%) and Africa (0.5%). Respondents primarily were paediatric or adult rheumatologists (85%) or had an interest in rheumatology (8%), but also included clinical academics (specialty not defined, 4%), dermatologists (0.5%), neurologists (0.5%), physiotherapists (1%) or other professionals (1%). The majority of respondents had substantial experience in the specialty (74% with ≥10 years of experience) and worked within paediatrics/mainly paediatrics (82.5% vs 17.5% of respondents working with adults). Responses were summarised as percentages of participants ranking items as critical for decision-making (score 7–9) for each item (clinical/research), shown in online supplementary table S1. Availability of investigations to clinicians within clinical practice was also summarised from responses received in Delphi 1 (online supplementary table S4 and online supplementary figure S1).

### Patient/parent surveys

In total, 301 surveys were completed (198 from parents, 103 patients). To allow time for sufficient data capture for parent/patient questionnaires, data collection continued after the consensus meeting. At the consensus meeting, data were available from 16 completed patient surveys and 22 parent surveys. Decisions made at the consensus meeting with 38 responses still held true in the final analysis of 301 replies. Responses were received from Europe (53%), North America (44%) and other continents (3%). Patients completing the questionnaire were a median of 15 years of age (IQR 12–17). Parents completed questionnaires for children who had a median age of 11 years (IQR 7–15). Overall, there was good agreement between patient/parent surveys and the healthcare professionals’ Delphi and items agreed at the consensus meeting (online supplementary table S1). Key exceptions are summarised in table 2.

**Table 2:** Key differences between opinions of patients/parents and healthcare professionals

Item	Patients’ opinion	Parents’ opinion	Healthcare professionals’ opinion	Outcome from consensus meeting	Comments/reasons for retaining in dataset
Raynaud’s phenomenon	Equivocal	Equivocal	Consensus in	Consensus in	Important for overlap phenotypes especially myositis–scleroderma
Use of an age-appropriate patient/parent measure of function	Equivocal	Equivocal	Consensus in	Consensus in	Retained (with the option of using alternative tools to allow for country-specific requirements) since these are standard outcome measures for research in JDM
Use of an age-appropriate patient/parent measure of quality of life	Equivocal	Equivocal	Consensus in	Consensus in	
Parent/patient global assessment VAS	Equivocal	Equivocal	Consensus in	Consensus in	
Physician global assessment VAS	Equivocal	Equivocal	Consensus in	Consensus in	
Fatigue due to myositis (within PROM)	Equivocal	Consensus in	Consensus in	Consensus in—as part of a PROM	Quantifiable outcome measure
Questions related to physiotherapy	Equivocal	Equivocal	Consensus in	Consensus in	Increasingly a defined therapeutic intervention; omitting would be akin to not asking about medicines
Pubertal assessment	Equivocal	Equivocal	(Not asked)*	Consensus in	Important outcomes of disease activity/damage/adverse effects of medication
Height of patient	Equivocal	Consensus in	Consensus in	Consensus in	
Weight of patient	Equivocal	Consensus in	Consensus in	Consensus in	
Items related to major organ involvement—cardiac/pulmonary/gastrointestinal	Equivocal	Consensus in	Consensus in	Consensus in	Important implications for disease severity, treatment and prognosis
Specific questions about pain	Consensus in	Consensus in	(Not asked)	Consensus out	Thought to be part of standard care (questions that would be asked by a clinician in a clinic consultation)
Specific questions about medicines	Consensus in	Consensus in	(Not asked)	Consensus out	
Irritability due to JDM	Equivocal	Consensus in	(Not asked)	Consensus out	Too non-specific and variable interpretation in different countries

*Added to patient/parent questionnaire after discussion in patient/parent focus groups.

JDM, juvenile dermatomyositis; PROM, patient/parent-reported outcome measure; VAS, Visual Analogue Scale.

### Consensus meeting and output

All invited experts (n=18) attended the consensus meeting (Liverpool, March 2015), representing Europe (n=10), North America (n=6), Latin America (n=1) and Asia (n=1). Specialties included paediatric rheumatology (n=13), adult rheumatology (n=2), paediatric dermatology (n=1), paediatric neurology (n=1) and physiotherapy (n=1). Parents/patients were not included. Output from the consensus meeting is shown in online supplementary table S1. A set of recommendations for first visit, for each visit and for annual assessment was made. Refinement took place following the consensus meeting via three rounds of SurveyMonkey, principally to better define myositis overlap features and disease damage items (shown in online supplementary table S1), with the same members of the expert group (100% response rate).

### Testing the dataset in practice

Glossaries of definitions/instructions to aid completion, along with muscle strength-testing sheets, were formulated into appendices, approved by the SC. Twenty clinicians tested the dataset (October 2016–April 2017); eight were present at the consensus meeting, three had completed the Delphi and nine were new to the dataset. Time taken to complete the dataset in clinical practice ranged from 5 to 45 min (median time 15 min). In addition, 15/20 (75%) found the dataset helpful in practice. Feedback was reviewed in detail by the SC and refinements made.

### Completed optimal dataset

The resulting optimal dataset is summarised within tables 3–5 representing three forms. They consist of 123 items: 12 (plus 6 items conditional on responses to the initial 12) within form A, to be completed at first/baseline data entry only; 56 (plus 20 conditional on responses to the 56) within form B, to be completed at every clinic visit representing status of the patient at the current time point; and 55 (plus 15 conditional on responses to the 55) within form C, to be completed at baseline and then annually to capture disease damage. The complete dataset with glossary of definitions and muscle strength-testing sheets can be found in the website of University of Liverpool (http://ctrc.liv.ac.uk/JDM/) and online supplementary table S5.

**Table 3:** Summary of items included in the JDM optimal dataset, form A (completed at first/baseline visit only)

Section heading	Items		Additional items conditional on previous response (summary)
Personal factors/demographics	1	Date of birth (year and month of birth±day of birth)	
	2	Sex of patient	
Diagnostic factors	3	Date (year and month) of first symptom of myositis	
	4	Date (year and month) of diagnosis of JDM	
	5	At the time of diagnosis did the patient have proximal muscle weakness?	
	6	At the time of diagnosis did the patient have typical skin features of JDM (Gottron’s/heliotrope)?	
	7	Was an MRI scan done at diagnosis?	Choice of options for MRI result (four options)
	8	Was a muscle biopsy done at diagnosis?	Choice of options for biopsy result (four options plus total biopsy score if available)
	9	Were myositis-specific antibodies tested at diagnosis?	If positive, asked to select all that apply (eight options)
	10	Were myositis-associated antibodies tested at diagnosis?	If positive, asked to select all that apply (nine options)
Treatments received prior to diagnosis of JDM	11	Did this patient receive systemic glucocorticoid prior to diagnosis of JDM?	If yes, asked to select all that apply (three options)
	12	Did this patient receive any synthetic or biologic disease modifying anti-rheumatic drug prior to the diagnosis of JDM?	If yes, asked to select all that apply (13 options)

JDM, juvenile dermatomyositis.

**Table 4:** Summary of items included in the JDM optimal dataset, form B (completed at every visit representing status of the patient at the current time point)

Section heading	Items		Additional items conditional on previous response (summary)
Growth	1	Height of patient (in centimetres)	
	2	Weight of patient (in kilograms)	
Muscular involvement	3	Presence of symmetrical proximal muscle weakness	
	4	Childhood Myositis Assessment Scale score	State score (out of 52)
	5	Manual Muscle Testing score	State score (out of 80)
	6	VAS score for global muscle disease activity	If measured, mark score on 10 cm line*
Skeletal involvement	7	Arthritis due to myositis	
	8	Joint contractures due to myositis	
	9	VAS score for global skeletal disease activity	If measured, mark score on 10 cm line*
Cutaneous involvement	10	Gottron’s papules or Gottron’s sign	
	11	Heliotrope rash	
	12	Periungual capillary loop changes (plus measure of capillary density if available)	
	13	Malar or facial erythema	
	14	Linear extensor erythema	
	15	‘V’ sign	
	16	Shawl sign	
	17	Non sun-exposed erythema	
	18	Extensive cutaneous erythema, which may include erythroderma	
	19	Livedo reticularis	
	20	Cutaneous ulceration	
	21	Mucus membrane lesions	
	22	Mechanic’s hands	
	23	Cuticular overgrowth	
	24	Subcutaneous oedema	
	25	Panniculitis	
	26	Alopecia (non-scarring)	
	27	Calcinosis (with active disease)	
	28	VAS score for global cutaneous disease activity	If measured, mark score on 10 cm line*
Features suggestive of myositis overlap	29	Does this patient have a myositis overlap condition?	If yes, asked to select all that apply (four options)
	30	Raynaud’s phenomenon	
	31	Sclerodactyly	
Gastrointestinal involvement	32	Dysphagia due to myositis	
	33	Abdominal pain due to myositis	
	34	Gastrointestinal ulceration due to myositis	
	35	VAS score for global gastrointestinal disease activity	If measured, mark score on 10 cm line*
Pulmonary involvement	36	Pulmonary involvement/respiratory muscle weakness or interstitial lung disease due to myositis	
	37	Dysphonia due to myositis	
	38	VAS score for global pulmonary disease activity	If measured, mark score on 10 cm line*
Cardiovascular involvement	39	Cardiovascular involvement due to myositis	
	40	BP recording	State systolic and diastolic measurement
	41	BP elevated suggesting hypertension (for age of patient)	
	42	VAS score for global cardiovascular disease activity	If measured, mark score on 10 cm line*
Constitutional features	43	Fever (>38°C) due to myositis	
	44	Weight loss (>5%) due to myositis	
	45	Fatigue due to myositis	
	46	VAS score for global constitutional disease activity	If measured, mark score on 10 cm line*
Global disease assessment by clinician	47	Physician VAS score of global disease activity	If measured, mark score on 10 cm line*
	48	Physician VAS score of extramuscular disease activity	If measured, mark score on 10 cm line*
Global disease assessment by patient/parent	49	Patient/parent VAS score for global disease activity	If measured, mark score on 10 cm line* and state who completed (four options)
	50	Patient/parent VAS score for pain	If measured, mark score on 10 cm line*
PROM	51	Use of an age-appropriate PROM of function	Asked to state PROM used and score
	52	Use of an age-appropriate patient/parent-reported measure of quality of life	Asked to state PROM used and score
Investigations	53	Elevation of any muscle enzyme (including CPK, LDH, aldolase, AST/SGOT, ALT/SGPT) above normal range	If elevated, asked to select which apply (five options)
Specimens available	54	Has this patient had specimens taken that may be available for specific research projects? This may include DNA, serum, biomarkers, biopsy tissue or other material	If answer is ‘yes’, asked to select which apply (three options)
Treatment	55	Is the patient on treatment (now or since last visit)?	Asked to select all that apply (16 options) and to state dose, route and frequency for each medication
	56	Is the patient doing a regular exercise routine prescribed by a healthcare professional aimed at improving/maintaining: (A) range of movement? (B) muscle strength?	

*0 is inactive or lowest score and 10 is most active or highest score on 10 cm VAS scores.

ALT, alanine transaminase; AST, aspartate aminotransferase; BP, blood pressure; CPK, creatine phosphokinase; JDM, juvenile dermatomyositis; LDH, lactate dehydrogenase; PROM, patient/parent-reported outcome measure; SGOT, serum glutamic oxaloacetic Transaminase; SGPT, serum glutamic-pyruvic transaminase; VAS, Visual Analogue Scale.

**Table 5:** Summary of items included in the JDM optimal dataset, form C (completed at baseline visit and then annual visits only)

Section heading	Items		Additional items conditional on previous response (summary)
Muscular damage items	1	Muscle atrophy (clinical)	
	2	Muscle weakness not attributable to active muscle disease	
	3	Muscle dysfunction: decrease in aerobic exercise capacity	
	4	VAS for global muscle disease damage	Mark score on 10 cm line*
Skeletal damage items	5	Joint contractures (due to myositis)	
	6	Osteoporosis with fracture or vertebral collapse (excluding avascular necrosis)	
	7	Avascular necrosis	
	8	Deforming arthropathy	
	9	VAS for global skeletal disease damage	Mark score on 10 cm line*
Cutaneous damage items	10	Calcinosis (persistent)	
	11	Alopecia (scarring)	
	12	Cutaneous scarring or atrophy (depressed scar or cutaneous atrophy)	
	13	Poikiloderma	
	14	Lipoatrophy/lipodystrophy	
	15	VAS for global cutaneous disease damage	Mark score on 10 cm line*
Gastrointestinal damage items	16	Dysphagia (persistent)	
	17	Gastrointestinal dysmotility, constipation, diarrhoea or abdominal pain (persistent)	
	18	Infarction or resection of bowel or other gastrointestinal organs	
	19	VAS for global gastrointestinal disease damage	Mark score on 10 cm line*
Pulmonary damage items	20	Dysphonia (persistent)	
	21	Impaired lung function due to respiratory muscle damage	
	22	Pulmonary fibrosis	
	23	Pulmonary hypertension	
	24	VAS for global pulmonary disease damage	Mark score on 10 cm line*
Cardiovascular damage items	25	Hypertension requiring treatment for >6 months	
	26	Ventricular dysfunction or cardiomyopathy	
	27	Assessed in adults (>18 years of age) only: angina or coronary artery bypass	
	28	Assessed in adults (>18 years of age) only: myocardial infarction	
	29	VAS for global cardiovascular damage	Mark score on 10 cm line*
Peripheral vascular damage items	30	Tissue or pulp loss	
	31	Digit loss or limb loss or resection	
	32	Venous or arterial thrombosis with swelling, ulceration or venous stasis	
	33	Assessed in adults (>18 years of age) only: claudication	
	34	VAS for global peripheral vascular disease damage	Mark score on 10 cm line*
Pubertal status of patient	35	Pubertal assessment completed by physician or by patient (self-assessment)	Tanner score (1–5)
Endocrine damage items	36	Growth failure	
	37	Delay in development of secondary sexual characteristics (>2 SD beyond mean for age)	
	38	Hirsutism or hypertrichosis	
	39	Irregular menses	
	40	Primary or secondary amenorrhoea	
	41	Diabetes mellitus	
	42	In adults (>18 years of age): infertility—male or female	
	43	In adults (>18 years of age): sexual dysfunction	
	44	VAS for global endocrine disease damage	Mark score on 10 cm line*
Ocular damage items	45	Cataract resulting in visual loss	
	46	Visual loss, other, not secondary to cataract	
	47	VAS for global ocular disease damage	Mark score on 10 cm line*
Infection damage items	48	Chronic infection	
	49	Multiple infections	
	50	VAS for global infection damage	Mark score on 10 cm line*
Malignancy	51	Presence of malignancy	
	52	VAS for malignancy (complications)	Mark score on 10 cm line*
Other damage	53	Death	Include cause and date of death
	54	VAS for any other damage	Mark score on 10 cm line* and add details of other damage
Global disease assessment damage	55	Physician VAS of global disease damage	Mark score on 10 cm line*

*0 is inactive or lowest score and 10 is most active or highest score on 10 cm VAS scores.

JDM, juvenile dermatomyositis; VAS, Visual Analogue Scale.

## Discussion

An internationally agreed JDM dataset has been designed for use within a clinical setting, with the potential to significantly enhance research collaboration and allow effective communication between groups. The accompanying glossary of definitions may be particularly helpful to those in training or physicians less familiar with JDM and for standardisation of the information. Key items are included within the dataset that allow documentation of disease activity and damage with the ability to measure change over time. If adopted widely, the dataset could enable analysis of the largest possible number of patients with JDM to improve disease understanding. It is anticipated that further ratification of the dataset will take place when incorporated into existing registries and national/international collaborative research efforts. It is acknowledged that updates may be needed in the future to incorporate advances in JDM.

When tested in practice by a small number of clinicians, the forms took between 5 and 45 min to complete. The wide range is likely to be due to some respondents interpreting this question as time taken to complete the actual forms, while others may have documented time taken to complete all the tasks within the forms, including clinical examination. It is likely that completion time will be reduced as clinicians become familiar with the questions over time and employment of electronic data entry systems. The dataset does not encompass every aspect of a clinic consultation. Other factors such as adverse effects to medication or details of pain (ranked important by patients/parents) should be covered as part of standard care.

This study has benefited from the enormous contribution of patients and parents. It is interesting that patients do not necessarily perceive items such as shortness of breath, chest pain and abdominal symptoms as important in JDM whereas for clinicians, major organ involvement has important implications for prognosis and treatment choices.^31–37^ Likewise, growth and pubertal parameters were rated less important by patients/parents but retained due to impact of active disease and corticosteroid treatment on growth.^38 39^ Self-assessment is allowable to make pubertal assessment more acceptable to patients.^40^ Notable discrepancies in healthcare professional and patient/parent opinion included the use of PROMs capturing function and health-related quality of life (HRQOL). The benefits and limitations of individual tools have been described.^22 27^ Within this study, comments from patient/parent surveys and focus groups suggested a dislike of 0–10 cm scales used in VAS measurements (data not shown). It is possible that a pain/general VAS is not adequate to capture the complexity of pain or overall feelings for a patient, particularly due to the variability of the disease. Despite this caveat, clinicians recognise the need to have outcome-driven data that include measures of activity, participation, pain and HRQOL.^27^ Patients with JDM have been found to have significant impairment in their HRQOL compared with healthy peers.^41^ PROMs used within the IMACS and PRINTO core sets, including the Childhood Health Assessment Questionnaire and Child Health Questionnaire, are not designed specifically for JDM but have been evaluated and endorsed for use in juvenile myositis.^22^ The Juvenile Dermatomyositis Multidimensional Assessment Report (JDMAR) is a multifunctional tool that includes function, quality of life, fatigue and adverse effects of medications that has been specifically developed for JDM.^23^ It is currently undergoing further validation. Fatigue, rated as important by parents in this work, is included within the JDMAR. During the consensus meeting, it was not possible to define a single agreed PROM for function (activity) or HRQOL (participation) despite taking into consideration results of the healthcare professionals’ Delphi, patient/parent surveys and feedback from patients within a UK focus group (online supplementary table S2). The difficulty of PROMs being internationally accepted was discussed and noted. Specifically, items within tools developed in Europe/North America may not be relevant in economically less developed countries. It was agreed that the dataset would include a recommendation to use ‘an age-appropriate patient/parent-reported outcome of function’ and ‘an age-appropriate patient/parent-reported measure of quality of life’. More work is needed to make PROMs acceptable to patients/parents and applicable to their disease.^42 43^

This study is limited by the fact that patient/parent questionnaires were available in English only, reducing the number of countries that could contribute; hence, there is low patient participation outside of Europe and the USA. Complete data from patient/parent surveys were not available at time of the consensus meeting. However, reanalysis of outcomes after the close of the patient/parent survey showed that decisions made at the consensus meeting still held. Initial response rate to Delphi 1 was low (estimated at 26% of potential specialty group membership). However, not all members of the respective organisations contacted would be expected to answer a paediatric-specific survey as described previously. Response rates and attrition between Delphi 1 and 2 were as expected from paediatric rheumatology studies with similar methodology.^44–46^ Despite inclusion of neurology and dermatology experts in the consensus meeting, the participants of this study were primarily rheumatologists.

Considerable discussion took place during the consensus meeting regarding the assessment of cutaneous disease in myositis. There are many tools available,^22^ but no single tool has been universally accepted. It can be difficult to define skin activity versus damage, particularly without a skin biopsy. After voting on individual skin items and comparing two tools endorsed in JDM, the abbreviated Cutaneous Assessment Tool (aCAT) and Disease Activity Score (DAS) skin score,^22^ agreement was reached to use items within the aCAT as disaggregated skin manifestations. These items are recognised to reflect cutaneous lesions associated with disease activity and damage in juvenile and adult myositis.^22^ Within the item ‘periungual capillary loop changes’, ‘measure of nailfold capillary density if available’ was added in recognition of nailfold density relating to prognosis.^47 48^ A direct comparison of all available skin tools was outside the remit of this study. Recent published work evaluating the Cutaneous Dermatomyositis Disease Area and Severity Index (CDASI) and the Cutaneous Assessment Tool Binary Method (CAT-BM) in JDM confirms the reliability of both tools when used by paediatric dermatologists or rheumatologists.^49^

The consensus-driven dataset developed in this study, like IMACS and PRINTO core sets, includes physician and patient/parent global activity, each of which is included in recently defined response criteria for minimal, moderate and major improvement in JDM.^8^ IMACS measures muscle strength using Manual Muscle Testing, whereas CMAS is used within the PRINTO core set. Both were retained in the consensus dataset. Both tools have been found to have very good inter-rater reliability (when summary scores are used)^22^ and either is allowed in the recently defined American College of Rheumatology/European League Against Rheumatism–approved response criteria.^8^ The overlap between the IMACS/PRINTO core sets and items contained within the consensus dataset is unsurprising as all core sets aim to capture and measure disease activity and damage over time. A key difference is that the consensus dataset does not use specific tools to record disease activity, such as the Myositis Disease Activity Assessment Tool or the DAS, but rather uses disaggregated items, each of which has been evaluated by a multistage consensus-driven process that considered value for both clinical use and research. The dataset was developed with a key aim for it to be incorporated into existing registries, allowing comparison of data between groups. The already available web-based Euromyositis registry, www.euromyositis.eu, is free to use in clinical practice and for research and includes a JDM proforma, which will be modified where needed to include items in this new dataset. Likewise, at the time of writing, the CARRA Registry is in the final stages of adding JDM (https://carragroup.org/) and will include the items contained in this consensus dataset. The JDCBS (https://www.juveniledermatomyositis.org.uk/) aims to incorporate this dataset as far as possible.

Research priorities defined during the consensus meeting included the need to further develop skin assessment tools that are practical within a busy clinical setting, develop an abbreviated muscle assessment tool that removes redundant items from a combined Childhood Myositis Assessment Scale and Manual Muscle Testing and to further develop PROMs so that they are applicable to JDM and acceptable to patients.

## Conclusion

Through a robust international consensus process, a consensus dataset for JDM has been formulated that can capture disease activity and damage over time. This dataset can be incorporated into national and international collaborative research efforts, including existing clinical research databases and used routinely while evaluating patients with JDM.
