# Supplementary material for: Development of a consensus core dataset in juvenile dermatomyositis for clinical use to inform research
Source: Ann Rheum Dis. 2017 Oct 30;77(2):241–50. doi: 10.1136/annrheumdis-2017-212141 (PMC5816738; doi:10.1136/annrheumdis-2017-212141)
Supplement: Supplementary file 1 [file annrheumdis-2017-212141supp001.docx]

**Supplementary Table S1: Results matrix showing consensus results for healthcare professional Delphi, patient / parent questionnaire and consensus meeting** (Items achieving consensus highlighted in bold; ‘total’ = numbers scoring 0-9 on a 1-9 scale for each item; items shown in italics conditional on response to previous question – only applicable if answer to preceding question is ‘yes’)

| **Section headings** | **Item defined in initial template [background work]** | **Item used in healthcare professional Delphi** | **Delphi 1: number / total (%) scoring 7-9 for clinical** | **Delphi 1: number / total (%) scoring 7-9 for research** | **Delphi 2: number / total (%) scoring 7-9 for clinical** | **Delphi 2: number / total (%) scoring 7-9 for research** | **Parent**  **Survey: number (%) scoring 3: very important** | **Patient Survey: number (%) scoring 3: very important** | **Consensus meeting: Number (%) voting for inclusion in dataset** | **Consensus in / out** | **Comments** |
| --- | --- | --- | --- | --- | --- | --- | --- | --- | --- | --- | --- |
| **Demographic** | Date of birth | Date of birth of patient (month / year +/- day) | 149 / 258 (58) | 172 / 260 (66) | **105 / 146 (72)** | **117 / 146 (80)** | N/A | N/A | **17 / 17 (100)** | **IN** | Option of not completing full date of birth, allowing for country specific ethics |
|  | Gender | Gender of patient | 136 / 258 (53) | 176 / 262 (68) | 93 / 146 (64) | **123 / 146 (84)** | N/A | N/A | **16 / 17 (94.1)** | **IN** | Changed to sex of patient after discussion at consensus meeting |
|  | - | Race of patient | 97 / 257 (38) | 153 / 258 (59) | 50 / 146 (34) | **110 / 145 (76)** | N/A | N/A | 11 / 17  (64.7) | OUT | Difficult to collect accurate data when race self-identified |
|  | Ethnicity | Ethnicity of patient | 81 / 258 (31) | 142 / 259 (55) | 40 / 146 (27) | **102 / 146 (70)** | N/A | N/A | 0 / 17  (0) | OUT | Difficult to collect accurate data when ethnicity self-identified |
| **Family history** | Family history of autoimmune disease | Family history of autoimmune disease | 121 / 257 (47) | 156 / 260 (60) | 64 / 146 (44) | **104 / 146 (71)** | N/A | N/A | 2 / 17  (11.8) | OUT | Importance recognised but poor reliability; difficult to capture robust data |
|  | Family history of neuromuscular disease | Family history of neuromuscular disease | 117 / 259 (45) | 130 / 258 (50) | 71 / 146 (49) | 88 / 145 (61) | N/A | N/A | 0 / 17 (0) | OUT | Importance recognised but poor reliability; difficult to capture robust data |
| **Disease type** | Disease type  (JDM / JPM / overlap / incomplete DM / IBM) | - | N/A | N/A | N/A | N/A | N/A | N/A | N/A | N/A |  |
| **Diagnostic data** | Date of first symptoms | Date (year / month) of first symptom of myositis | 149 / 236 (63) | **182 / 236 (77)** | **107 / 145 (74)** | **127 / 145 (88)** | N/A | N/A | **17 / 17 (100)** | **IN** |  |
|  | Date of diagnosis | Date (year / month) of diagnosis of JDM | 157 / 236 (67) | **188 / 226 (83)** | **113 / 145 (78)** | **129 / 145 (89)** | N/A | N/A | **17 / 17 (100)** | **IN** |  |
| Diagnostic data - muscle | Symmetrical proximal muscle weakness | Presence of symmetrical muscle weakness at diagnosis | **207 / 232 (89)** | **206 / 236 (87)** | **140 / 145 (97)** | **135 / 145 (93)** | Asking if your child has any weakness  **172 /197 (87)** | Asking if you have any weakness  **82 / 103 (80) IN** | **17 / 17 (100)** | **IN*** | *Combined with same question at every visit ie. Moved to section 2 (form B) rather than been counted as separate outcomes. |
|  | - | Use of a validated tool to score muscle strength / function at diagnosis | 154 / 235  (66) | **215 / 237 (91)** | **112 / 145 (77)** | **139 / 145 (96)** | Testing to see how strong your child is  **172 / 197 (87)** | Testing to see how strong you are  **81 / 103 (79) IN** | **16 / 17 (94.1)** | **IN*** |  |
|  | *-* | *Childhood myositis assessment scale (CMAS) at diagnosis* | 144 / 232  (62) | **204 / 233 (88)** | **101 / 141 (72)** | **138 / 142 (97)** |  |  | **16 / 17 (94.1)** on 2^nd^ vote | **IN*** |  |
|  | *-* | *Manual muscle testing score (MMT8) at diagnosis* | 138 / 226 (61) | **204 / 233 (88)** | **100 / 143 (70)** | **138 / 142 (97)** |  |  | **16 / 17 (94.1)** | **IN*** |  |
| Diagnostic data - skin:  Dermatological features of JDM | Heliotrope rash | Presence of heliotrope rash at diagnosis | **204 / 233**  **(88)** | **197 / 234 (84)** | **137 / 145 (94)** | **136 / 145 (94)** | Asking if your child has any skin rashes  **173 / 196 (88)** | Asking if you have any skin rashes  **72 / 102 (71)** | **17 / 17 (100)** | **IN*** | *Combined with same question at every visit ie. Moved to section 2 (form B) rather than been counted as separate outcomes at time of diagnosis.  Decision made after extensive discussion at consensus meeting to use variables included in abbreviated CAT tool with each item in activity section of tool (n=17) separated into individual outcomes in section 2 (form B) + calcinosis + VAS (total =19). In addition, 6 cutaneous items from MDI tool to be included in section 3 (form C) representing damage. |
|  | Gottron’s papules or Gottron’s sign | Presence of Gottron's papules / Gottron's sign at diagnosis | **209 / 233 (90)** | **197 / 234 (84)** | **137 / 145 (94)** | **136 / 145 (94)** |  |  | **17 / 17 (100)** | **IN*** |  |
|  | Nailfold capillary changes | Presence of nail-fold capillary changes at diagnosis | **183 / 234**  **(78)** | **189 / 234 (81)** | **123 / 144 (85)** | **132 / 145 (91)** |  |  | **16 / 17 (94.1)** | **IN*** |  |
|  | Other characteristic rash of JDM | Presence of other JDM rash at diagnosis | **169 / 234**  **(72)** | **171 / 234 (73)** | **124 / 145 (86)** | **125 / 145 (86)** |  |  | **16 / 17 (94.1)** | **IN*** |  |
|  | - | Use of a validated skin tool at diagnosis of JDM | 85 / 226  (38) | **162 / 224 (72)** | 52 / 141 (37) | **125 / 141 (89)** | Looking for rashes / skin signs that may suggest active JDM  **187 / 197 (95)** | Looking for rashes / skin signs that may suggest active JDM  **83 / 102 (81)** | **14 / 17 (82.4)** | **IN*** |  |
|  | *-* | *Use of the Cutaneous Assessment Tool (CAT) at JDM diagnosis* | 77 / 198 (39) | **147 / 199 (74)** | 44 / 134 (33) | **115 / 135 (85)** |  |  | 1 / 17 (5.9)  Re-vote for abbreviated CAT tool **17 / 18** **(94.4)** | **IN*** |  |
|  | *-* | *Use of Disease Activity Index (DAS) at JDM diagnosis* | 103 / 206  (50) | **165 / 207 (80)** | 63 / 139 (45) | **129 / 140 (92)** |  |  | 3 / 17 (17.6) | OUT |  |
| Diagnostic data- investigations | Muscle biopsy evidence of myositis | Muscle biopsy evidence of myositis @ diagnosis | 60 / 213 (28) | 112 / 210 (53) | 29 / 142 (20) | 79 / 141 (56) | N/A | N/A | **17 / 17 (100)** | **IN** | If muscle biopsy done |
|  | Elevation of muscle enzymes | Elevation of muscle enzymes | **185 / 213 (87)** | **187 / 210 (89)** | **135 / 142 (95)** | **135 / 142 (95)** | Taking blood tests to monitor how active the disease is  **177 / 197 (90)** | Taking blood tests to monitor how active the disease is  **81 / 101 (80)** | **17 / 17 (100)** | **IN** | Combined with same question at every visit as one outcome |
|  | *CPK* | *Creatine phosphokinase (CK/CPK) above normal range* | **159 / 212 (75)** | **168 / 208 (81)** | **118 / 142 (83)** | **123 / 142 (87)** |  |  | **15 / 17 (88.2)** | **IN** | Combined with same question at every visit as one outcome |
|  | *LDH* | *Lactate dehydrogenase (LDH/LD) above normal range* | 127 / 211  (60) | 137 / 205 (67) | 92 / 142 (65) | **106 / 142 (75)** |  |  | 11 / 17 (64.7)  Re-vote combining all muscle enzymes  **16 (94.1)** | **IN** (as drop down option) | Combined with same question at every visit as one outcome (drop down box option to choose which muscle enzymes elevated if answer to ‘elevation of muscle enzymes’ is ‘yes’) |
|  | *Aldolase* | *Aldolase above*  *normal range* | 110 / 200  (55) | 127 / 197 (64) | 77 / 136 (57) | 93 / 137 (68) |  |  | 4 / 17 (23.5)  Re-vote combining all muscle enzymes  **16 / 17 (94.1)** |  |  |
|  | *AST / SGOT* | *Aspartate Aminotransferase (AST) / Serum Glutamic Oxaloacetic Transaminase (SGOT) above normal range* | 119 / 210  (57) | 131 / 207 (63) | 84 / 142 (59) | **102 / 142 (72)** |  |  | 13 /17 (76.5)  Re-vote combining all muscle enzymes  **16 / 17 (94.1)** |  |  |
|  | *ALT / SGPT* | *Alanine transaminase (ALT) / Serum glutamic-pyruvic transaminase (SGPT) above normal range* | 103 / 209  (49) | 120 / 208 (58) | 67 / 141 (48) | 90 / 141 (64) |  |  | 3 / 17 (17.6)  Re-vote combining all muscle enzymes  **16 / 17 (94.1)** |  |  |
|  | EMG changes of myopathy | Electro-myography (EMG) changes of myositis at diagnosis | 56 / 212 (26) | 67 / 208 (32) | 31 / 142 (22) | 40 / 142 (28) | N/A | N/A | 9 / 17 (52.9) | OUT |  |
|  | MRI changes of myositis | Magnetic resonance imaging (MRI) changes of myositis at diagnosis | 143 / 212 (67) | 159 / 205 (78) | **102 / 141 (72)** | **117 / 141 (83)** | Asking your child to have a scan such as an MRI scan of their muscles to monitor disease  **132 / 188 (70)** | Asking you to have a scan such as an MRI scan of your muscles  **70 / 98 (71)** | **17 / 17 (100)** | **IN** |  |
|  | Autoantibody positivity: Option of selecting ANA and individual myositis specific (MSA) or myositis associated (MAA) auto-antibodies (27 options) | Antinuclear antibody (ANA) positivity at diagnosis | 49 / 212 (23) | 91 / 210 (43) | 25 / 142 (18) | 72 / 142 (51) | N/A | N/A | 9 / 17  (52.9) | OUT | Non-specific; not useful for diagnosis or as a biomarker. |
|  |  | Myositis specific antibody (MSA) positive at diagnosis | 81 / 207 (39) | 129 / 202 (64) | 47 / 138 (34) | **106 / 139 (76)** | N/A | N/A | **17 / 17 (100)** | **IN** | Increasingly more readily available and felt to be critical for future care and phenotype of disease. |
|  |  | Myositis associated antibody (MAA) positivity at diagnosis | 73 / 209 (35) | 125 / 202 (62) | 39 / 141 (28) | **105 / 140 (75)** | N/A | N/A | **17 / 17 (100)** | **IN** |  |
| *Additional questions added by steering committee in healthcare professional survey* | - | How important do you rate storing specimens @ diagnosis for other biomarkers? | 53 / 204 (26) | **154 / 195 (79)** | 25 / 139 (18) | **124 / 138 (90)** | N/A | N/A | **18 / 18 (100)** | **IN*** | *Merged into one question at first and every visit asking ‘Has this patient had specimens taken that may be available for research projects? This may include DNA, serum, biomarkers, biopsy tissue to other material’ (asked to specify which specimens available if answer to this question is yes). |
|  | *-* | *How important do you rate storing DNA @ diagnosis?* | 45 / 199 (23) | **137 / 109**  **(72)** | 25 / 139 (18) | **114 / 137 (83)** | N/A | N/A | **18 / 18 (100)** | **IN*** |  |
|  | *-* | *How important do you rate storing serum @ diagnosis?* | 52 / 199 (26) | **143 / 192**  **(74)** | 24 / 141 (17) | **120 / 142 (85)** | N/A | N/A | **18 / 18 (100)** | **IN*** |  |
|  | *-* | *Any other material you think should be stored at diagnosis?* | 21 / 133 (16) | 72 / 122  (59) | 9 / 95  (9) | **65 / 92 (71)** | N/A | N/A | 18 / 18 (100) | **IN*** |  |
| *Additional questions for parents / patients added by steering committee* | - | Importance of collecting the same information on all children / young people with JDM | N/A | N/A | N/A | N/A | **173 / 212 (82)** | 68 / 106 (64) | No vote | N/A | Underpins reason for study but not applicable to an individual outcome |
|  | - | Importance of storing information on all children / young people with JDM | N/A | N/A | N/A | N/A | **173 / 212 (82)** | 72 / 104 (69) | No vote | N/A |  |
| **Treatments given prior to diagnosis** | Treatments given prior to diagnosis of myositis / registration into database | Glucocorticoids given before diagnosis of JDM? | 135 / 202 (67) | **152 / 198**  **(77)** | **121 / 141 (86)** | **126 / 140 (90)** | N/A | N/A | 18 / 18 (100) | **IN** | Refined to ‘systemic glucocorticoids’ at consensus meeting. |
|  | *Steroid treatment – oral* | *Oral glucocorticoids prior to diagnosis?* | 120 / 183 (66) | **136 / 179 (76)** | **121 / 141 (86)** | **126 / 140 (90)** | N/A | N/A | 0 / 18 (0)  Re-vote to combine in drop-down box = **18 / 18 (100)** | **IN*** | *Combined into one outcome for ‘systemic glucocorticoids’ with drop down box option to choose to following options if systemic corticosteroids given: Oral / Intravenous / Intramuscular. |
|  | *Steroid treatment – intravenous* | *Intravenous glucocorticoids prior to diagnosis?* | **142 / 187 (76)** | **152 / 185 (82)** | **124 / 140 (89)** | **125 / 139 (90)** | N/A | N/A | 1 / 18 (5.6)  Re-vote to combine in drop-down box = **18 / 18 (100)** | **IN*** |  |
|  | *Steroid treatment – intra-muscular* | *Intra-muscular glucocorticoids prior to diagnosis?* | 111 / 181 (61) | **126 / 181 (70)** | **105 / 133 (79)** | **107 / 134 (80)** | N/A | N/A | 0 / 18 (0)  Re-vote to combine in drop-down box = **18 / 18 (100)** | **IN*** |  |
|  | *-* | *Intra-articular glucocorticoids prior to diagnosis?* | 66 / 195  (36) | 85 / 185 (46) | 58 / 140 (41) | 75 / 140 (54) | N/A | N/A | 0 (0) | OUT |  |
|  | *Steroid treatment – topical* | *Topical glucocorticoids prior to diagnosis?* | 61 / 188  (32) | 74 / 186  (40) | 46 / 140 (33) | 62 / 140 (44) | N/A | N/A | 0 (0) | OUT |  |
|  | Methotrexate / other Disease Modifying Anti-Rheumatic Drug (DMARD) | Any disease modifying anti-rheumatic drug given before diagnosis of JDM? | 140 / 202  (69) | **152 / 199 (76)** | **119 / 140 (85)** | **125 / 140 (89)** | N/A | N/A | **17 / 18 (94.4)** | **IN*** | *Combined into one outcome ‘any synthetic or biologic disease modifying anti-rheumatic drug (DMARD)’. If answer is ‘yes’, drop down boxes available for synthetic / biologic DMARDS. |
|  | Biologic | Any biologic given before the diagnosis of JDM? | **145 / 199**  **(73)** | **160 / 198 (81)** | **122 / 139 (88)** | **101 / 140 (92)** | N/A | N/A | 14 / 18 (77.8)  Re-vote as drop down option: **16 / 17 (94.1)** | **IN*** |  |
|  | - | Physiotherapy and/or occupational therapy before diagnosis of JDM? | 53 / 202 (26) | 65 / 199 (33) | 32 / 141 (23) | 43 / 141 (30) | N/A | N/A | 0 / 17 (0) | OUT |  |
| **Growth** | Height (cm) | Height of patient (cm) | **139 / 191 (73)** | **138 / 189 (73)** | **120 / 139 (86)** | **119 / 138 (86)** | Checking how well your child is growing (height)  **139 / 195 (71)** | Checking how well you are growing  44 / 97 (45) | **16 / 18** **(88.9)** after re-vote | **IN** |  |
|  | Weight (kg) | Weight of patient (kg) | **149 / 191 (78)** | **139 / 189 (74)** | **124 / 139 (89)** | **118 / 138 (86)** | Asking about any weight loss and checking your child’s weight  **144 / 197 (73)** | Asking about any weight loss and checking your weight  45 / 100 (45) | **15 / 18** **(83.3)** after re-vote | **IN** | On a regular basis |
| Additional variable on patient / parent questionnaire – added after focus groups |  | Puberty | N/A | N/A | N/A | N/A | Asking about how your child is developing or how puberty is progressing 122 / 190 (64) | Asking about how you are developing or how puberty is progressing  40 / 95 (42) | **16 / 18 (88.8)**  **Self – reported puberty 14/15 (93.3)** | **IN** | Self-assessment allowable |
| **Presence of active muscle disease** | Proximal muscle weakness | Presence of symmetrical proximal muscle weakness | **180 / 190 (95)** | **178 / 189 (94)** | **137 / 139 (99)** | **135 / 139 (97)** | Asking if your child has any weakness  **172 / 197 (87)** | Asking if you have any weakness  **82 / 103 (80)** | **18 / 18 (100)** | **IN** |  |
|  | Other muscle weakness | Presence of other muscle weakness | **162 / 191 (85)** | **163 / 189 (86)** | **135 / 139 (97)** | **135 / 139 (97)** |  |  | 1 / 18 (5.6) | OUT | The presence of other muscle weakness is assessed as part of MMT8 / CMAS assessment. Recognised that the perception of muscle weakness is a separate issue but not thought necessary to include. |
|  | - | Use of a validated tool to score muscle strength / function | 127 / 191 (66) | **170 / 190**  **(89)** | **108 / 139 (78)** | **135 / 139 (97)** | Testing to see how strong your child is  **172 / 197 (87)** | Testing to see how strong you are  **81 / 103 (79)** | **18 / 18 (100)** | **IN** |  |
|  | *CMAS* | *Childhood myositis assessment scale (CMAS)* | 109 / 188 (58) | **157 /188 (84)** | 91 / 137 (66) | **132 / 138 (96)** |  |  | **16 / 18 (88.9)** | **IN** |  |
|  | *MMT8* | *Manual muscle testing score (MMT8)* | 106 / 184 (58) | **142 / 184 (77)** | 91 / 138 (66) | **121 / 138 (88)** |  |  | **17 / 18 (94.4)** | **IN** |  |
| **Presence of active skin disease** | Heliotrope rash | Heliotrope rash present | **171 / 188 (91)** | **167 / 188 (89)** | **135 / 139 (97)** | **132 / 138 (96)** | Asking if your child has any skin rashes  **173 / 196 (88)** | Asking if you have any skin rashes  **72 / 102 (71)** | **17 / 18 (94.4)** | **IN** | Decision made after extensive discussion at consensus meeting to use variables included in abbreviated CAT tool with each item in activity section of tool (n=17) separated into individual outcomes in section 2 (form B) + calcinosis + VAS (total =19). In addition, 6 cutaneous items from MDI tool to be included in section 3 (form C) representing damage. Since photosensitivity not included in CAT, decision made to omit this question; felt to be difficult to assess and not necessarily important. |
|  | Gottron's papules or Gottron's sign | Gottron's papules or Gottron's sign | **169 /188 (90)** | **165 / 188 (88)** | **135 / 139 (97)** | **133 / 138 (96)** |  |  | **17 / 18 (94.4)** | **IN** |  |
|  | Nailfold capillary changes | Nailfold capillary changes | **137 / 188**  **(73)** | **156 / 190 (82)** | **118 / 139 (85)** | **126 / 139 (91)** |  |  | **17 / 18 (94.4)** | **IN** |  |
|  | Lipodystrophy | Lipodystrophy | **135 / 188 (72)** | **152 / 188 (81)** | **120 / 139 (86)** | **126 / 139 (91)** |  |  | **18 / 18 (100)** | **IN** |  |
|  | Calcinosis | Calcinosis | **168 / 188 (89)** | **175 / 188 (93)** | **135 / 139 (97)** | **135 / 139 (97)** |  |  | **18 / 18 (100)** | **IN** |  |
|  | Cutaneous ulceration due to myositis | Cutaneous ulceration due to myositis | **164 / 188 (87)** | **169 / 188 (90)** | **134 / 139 (96)** | **132 / 139 (95)** |  |  | **18 / 18 (100)** | **IN** |  |
|  | Subcutaneous oedema due to myositis: | Subcutaneous oedema due to myositis | **138 / 188 (73)** | **149 / 188 (79)** | **120 / 139 (86)** | **126 / 139 (91)** |  |  | **15 / 18 (83.3)** | **IN** |  |
|  | *(i) Generalised* |  |  |  |  |  |  |  |  |  |  |
|  | *(ii) Peri-orbital* |  |  |  |  |  |  |  |  |  |  |
|  | Malar / facial erythema due to myositis | Malar / facial erythema due to myositis | **142 / 188 (76)** | **151 / 188 (80)** | **121 / 137 (88)** | **126 / 138 (91)** |  |  | 10 (68.8)  Re-vote as part of CAT  **17 / 18 (94.4)** | **IN** |  |
|  | Shawl sign / V-sign due to myositis | Shawl sign / V-sign due to myositis | 120 / 186 (65) | **137 / 186 (74)** | **110 / 139 (79)** | **122 / 139 (88)** |  |  | 8 (50)  Re-vote as part of CAT  **17 / 18 (94.4)** | **IN** |  |
|  | Mechanic's hand | Mechanic's hand | 116 / 185 (63) | **134 / 184 (73)** | **106 / 138 (77)** | **120 / 138 (87)** |  |  | 4 (61.1)  Re-vote as part of CAT  **17 / 18 (94.4)** | **IN** |  |
|  | Alopecia due to myositis | Alopecia due to myositis | 96 / 187 (51) | 110 / 187 (59) | 82 / 138 (59) | **98 / 138 (71)** |  |  | 3 (17.6)  Re-vote as part of CAT  **17 / 18 (94.4)** | **IN** |  |
|  | Vasculopathic lesions due to myositis | Vasculopathic lesions due to myositis | **146 / 188 (78)** | **153 / 189 (81)** | **124 / 138 (90)** | **125 / 138 (91)** |  |  | **17 / 18 (94.4)** | **IN** |  |
|  | Photo-sensitivity due to myositis | Photo-sensitivity due to myositis | 110 / 187 (59) | **130 / 187 (70)** | **97 / 138 (70)** | **112 / 138 (81)** |  |  | 6 / 18 (33.3) | OUT |  |
|  | Livedo reticularis due to myositis | Livedo reticularis due to myositis | 103 / 186 (55) | 119 / 186 (64) | 92 / 138 (67) | **98 / 138 (71)** |  |  | 0 (0)  Re-vote as part of CAT  **17 / 18 (94.4)** | **IN** |  |
|  | Other erythema due to myositis | Other erythema due to myositis | 90 / 186 (48) | 110 / 186 (59) | 77 / 137 (56) | 92 / 137 (67) |  |  | 0 (0)  Re-vote as part of CAT  **17 / 18 (94.4)** | **IN** |  |
|  | Panniculitis due to myositis | Panniculitis due to myositis | 110 / 187  (59) | **134 / 186 (72)** | 93 / 138 (67) | **115 / 138 (83)** |  |  | 0 (0)  Re-vote as part of CAT  **17 / 18 (94.4)** | **IN** |  |
|  | - | Use of validated skin tool at every visit | 63 / 182 (35) | **128 / 184 (70)** | 39 / 132 (30) | **110 / 135 (81)** | Looking for rashes / skin signs that may suggest active JDM  **187 / 197 (95)** | Looking for rashes / skin signs that may suggest active JDM  **83 / 102 (81)** | **16 / 17 (94.1)** | **IN** |  |
|  | *-* | *Use of cutaneous assessment tool (CAT) at every visit* | 48 / 153 (31) | **115 / 157 (73)** | 27 / 125 (22) | **100 / 129 (78)** |  |  | **17 / 18 (94.4)** | **IN** | Variables within Abbreviated CAT to be used as outcomes |
|  | *-* | *Use of disease activity score (DAS) at every visit* | 76 / 162 (47) | **130 / 164 (79)** | 57 / 132 (43) | **114 / 133 (86)** |  |  | 0 / 17 (0) | OUT |  |
| **Major organ involvement** | Musculo-skeletal involvement due to myositis | Musculo-skeletal involvement due to myositis | **159 / 179 (89)** | **161 / 180 (89)** | **133 / 137 (97)** | **132 / 136 (97)** | Asking if your child has any muscle / joint pains  **177 / 197 (90)** | Asking if you have any muscle / joint pains  **81 / 102 (79)** | **16 / 17 (94.1)** | **IN** | Changed to skeletal involvement and moved to follow muscular involvement |
|  | *Arthritis* | *Arthritis* | **143 / 179 (80)** | **153 / 179 (85)** | **125 / 137 (91)** | **128 / 136 (94)** | Looking for swelling in any of the joints  **168 / 196 (86)** | Looking for swelling in any of the joints  **73 / 101 (72)** | **17 / 17 (100)** | **IN** |  |
|  | *Contractures due to myositis* | *Contractures due to myositis* | **140 / 179 (78)** | **147 / 179 (82)** | **130 / 137 (95)** | **130 / 136 (96)** | N/A | N/A | **17 / 17 (100)** | **IN** |  |
|  | Gastro-intestinal involvement due to myositis | Gastro-intestinal involvement due to myositis | **157 / 180 (87)** | **158 / 180 (88)** | **127 / 137 (93)** | **128 / 136 (94)** | Asking if your child has any difficulty with swallowing / eating or if they have tummy pain  **162 / 195 (83)** | Asking if you have any difficulty with eating or if you have any tummy pain  49 / 99 (49) | **17 / 17 (100)** | **IN** |  |
|  | *Dysphagia* | *Dysphagia* | **158 / 180 (88)** | **157 / 180 (87)** | **134 / 137 (98)** | **130 / 136 (96)** |  |  | **17 / 17 (100)** | **IN** |  |
|  | *Abdominal pain or GI ulceration due to myositis (exclude peptic ulcer disease)* | *Abdominal pain or GI ulceration due to myositis (exclude peptic ulcer disease)* | **150 / 180 (83)** | **153 / 180 (85)** | **128 / 136 (94)** | **125 / 135 (93)** |  |  | **14 / 17 (82.3)**  Re-vote for abdominal pain:  **16 / 17 (94.1)** | **IN** | Separated into 2 separate outcomes in consensus meeting: (1). Abdominal pain  (2). GI ulceration |
|  |  | *Gastro-intestinal ulceration due to myositis* | N/A | N/A | N/A | N/A | N/A | N/A | **17 / 17 (100)** | **IN** | Added as a separate item at consensus meeting (rather than combined with abdominal pain) |
|  | Respiratory involvement due to myositis: | Pulmonary involvement due to myositis suggesting interstitial lung disease | **166 / 180 (92)** | **161 / 180 (89)** | **132 / 136 (97)** | **131 / 136 (96)** | Asking if your child has any shortness of breath or chest pain  **150 / 191**  **(79)** | Asking if you have any shortness of breath or chest pain  63 / 100 (63) | **17 / 17 (100)** | **IN** |  |
|  | *(i) interstitial lung disease* |  |  |  |  |  |  |  |  |  |  |
|  | *(ii) Dysphonia* | *Dysphonia* | **153 / 181 (85)** | **152 / 180 (84)** | **130 / 137 (95)** | **127 / 136 (93)** |  |  | **17 / 17 (100)** | **IN** |  |
|  | Cardiac involvement due to myositis | Cardiac involvement due to myositis | **156 / 179 (87)** | **160 / 179 (89)** | **131 / 137 (96)** | **130 / 137 (95)** |  |  | **17 / 17 (100)** | **IN** | Domain name changed to cardio-vascular |
| Additional vote at consensus meeting | - | Blood pressure recording and  Blood pressure elevated suggesting hypertension | N/A | N/A | N/A | N/A | N/A | N/A | **12 / 15 (80)** | **IN** | As 2 separate outcomes |
| **Constitutional symptoms due to myositis** | Fever (>38 degrees C) due to myositis | Fever (>38 degrees C) due to myositis | **132 / 180 (73)** | **132 / 180 (73)** | **105 / 137 (77)** | **111 / 137 (81)** | N/A | N/A | **17 / 17 (100)** | **IN** |  |
|  | Weight loss (>5%) due to myositis | Weight loss (>5%) due to myositis | **140 / 179 (78)** | **130 / 180 (72)** | **123 / 137 (90)** | **123 / 137 (90)** | **144 / 197 (73)** | 45 / 100 (45) | **14 / 17 (82.4)** | **IN** |  |
|  | Fatigue due to myositis | Fatigue due to myositis | 116 / 179 (65) | 117 / 180 (65) | **105 / 137 (77)** | **111 / 137 (81)** | Asking if your child feels tired due to JDM  **153 /194 (79)** | Asking if you feel tired due to JDM  67 / 102 (66) | **17 / 17 (100)** | **IN** |  |
| Additional question added on patient / parent questionnaire after focus groups | - | Fatigue scale (parent/ patients) | N/A | N/A | N/A | N/A | Asking how tired your child feels using a formal ‘fatigue scale’  **133 / 191 (70)** | Asking how tired you feel using a formal ‘fatigue scale’  57 / 97 (59) | No vote | OUT | Discussion suggested that a fatigue scale could be incorporated into a PROM |
|  | Irritability due to myositis | Irritability due to myositis | 89 / 180 (49) | 94 / 180 (52) | 65 / 137 (47) | 78 / 137 (57) | Asking if your child feels irritable or miserable due to JDM  **142 / 195 (72)** | Asking if you feel irritable or miserable due to JDM  53 / 102 (52) | 0 / 17 (0) | OUT |  |
| **Overlap features** | Raynaud's phenomenon | Raynaud's phenomenon | 100 / 181 (55) | 117 / 180 (65) | **95 / 136 (70)** | **110 / 136 (81)** | Asking if your child has colour changes in their hands in cold weather  118 / 178 (66) | Asking if you get colour changes in your hands in cold weather  51 / 99  (52) | **16 / 17 (94.1)** | **IN** | Moved to separate section for features of myositis overlap |
| Additional vote from consensus meeting experts post meeting via survey monkey | - | Sclerodactyly | N/A | N/A | N/A | N/A | N/A | N/A | **15 / 18 (83.3)** | **IN** | Defined post meeting in survey monkey |
|  | - | Does this patient have features of a disease overlap | N/A | N/A | N/A | N/A | N/A | N/A | **17 / 18 (94.4 )** | **IN** |  |
|  | - | Swollen fingers | N/A | N/A | N/A | N/A | N/A | N/A | 11 / 18 (61) | OUT |  |
|  | - | Telangiectasia | N/A | N/A | N/A | N/A | N/A | N/A | 12 / 18 (66) | OUT |  |
|  | - | Digital infracts | N/A | N/A | N/A | N/A | N/A | N/A | 12 / 18 (66) | OUT |  |
| ADDITIONAL VOTE at consensus meeting |  | TO ADD a VAS score for all domains that represent disease activity, using the MYOACT domains | N/A | N/A | N/A | N/A | N/A | N/A | **17 / 17 (100)** | **IN** | Consequence = adding an additional 7 outcomes in section 2 (form B). |
| **Other** | Malignancy | Presence of malignancy | **130 / 172 (76)** | **142 / 177 (80)** | **122 / 136 (90)** | **126 / 137 (92)** | N/A | N/A | **15 / 15 (100)** | **IN** |  |
| Follow up | On-going follow up at this centre | Patient having on-going follow-up at this centre? | **126 / 168 (75)** | **140 / 171 (82)** | **115 / 131 (88)** | **120 / 132 (91)** | N/A | N/A | 6 / 15 (40) | OUT |  |
|  | *If NO:*  *(i) patient died (including cause of death and if due to myositis)* | *If NO: patient died (including cause of death and if due to myositis)* | **139 / 160 (87)** | **147 / 162 (91)** | **130 / 134 (97)** | **131 / 135 (97)** | N/A | N/A | **14 / 15 (93.3)** | **IN** | Changed to ‘death due to myositis’ – moved to damage section & included in damage variables |
|  | *(ii) Transfer to another geographical region* | *If NO: Transfer to another geographical region* | 108 / 163 66 | **121 / 163 (74)** | **106 / 133 (80)** | **112 / 134 (84)** | N/A | N/A | Not voted – N/A since stem question on on-going follow up voted out | OUT |  |
|  | *(iii) Transfer to adult care* | *if NO: Transfer to adult care* | **114 / 162 (70)** | **126 / 163 (77)** | **108 / 132 (82)** | **116 / 133 (87)** | N/A | N/A |  | OUT |  |
|  | *(iv) Patient in stable remission* | *Patient in stable remission* | **138 / 172 (80)** | **153 / 176 (87)** | **127 / 135 (94)** | **127 / 136 (93)** | N/A | N/A |  | OUT |  |
| **Global disease assessment by clinician** | Physician VAS – global activity (overall) | Use of physician scored measure of global disease activity | **125 / 177 (71)** | **158 / 180 (88)** | **118 / 136 (87)** | **128 / 136 (94)** | Asking your doctor to mark on a 0-10cm scale how well or unwell they think your child has been based on what you tell them and what they see at your clinic visit  119 / 193 (62) | Asking your doctor to mark on a 0-10cm scale how well or unwell they think you have been  38 / 97 (39) | Not scored (answered by next question) | **N/A** |  |
|  |  | *Physician VAS scale of global activity* | **121 / 174 (70)** | **157 / 177 (89)** | **114 / 137 (83)** | **130 / 137 (95)** |  |  | **18 / 100 (100)** | **IN** |  |
|  | Physician VAS – for extra-muscular activity | *Physician VAS scale for muscular activity* | 110 / 174 (63) | **149 / 177 (84)** | **106 / 137 (77)** | **127 / 137 (93)** |  |  | **18 / 100 (100)** | **IN** |  |
| **Global assessment by parent / patient** | - | Use of a patient/parent reported outcome measure of disease activity or function | 81 / 170 (48) | **134 / 170 (79)** | 64 / 137 (47) | **127 / 138 (92)** | Asking you or your child to complete a questionnaire that asks how easy or difficult it is for them to do things like get dressed, have a bath, do activities 118 / 193 (61) | Asking you to complete a list of questions that look at how easy or difficult it is for you to do things like get dressed, have a bath, do activities  52 / 101  (51) | **18 / 100 (100)** | **IN** |  |
|  | Parental / patient VAS | *Patient/parent VAS* | 89 / 171 (52) | **134 / 170 (79)** | 72 / 137 (53) | **128 / 137 (93)** | Asking you / your child to mark on a 0-10cm scale how well or unwell they have been over the last 4 weeks due to JDM  116 / 194 (60) | Asking you to mark on a 0-10cm scale how well or unwell you have been over the last 4 weeks due to JDM  48 / 100 (48) | **18 / 100 (100)** | **IN** |  |
| Additional question asked on patient / parent questionnaire  after discussion in focus groups | *-* | *Pain VAS (parent/ patient)* | N/A | N/A | N/A | N/A | Asking you / your child to rate on a scale of 0-10cm how much pain your child has had in the last 4 weeks due to JDM  123 / 195 (63) | Asking you to mark on a 0-10cm scale how much pain you have had over the last 4 weeks due to JDM  50 / 102  (49) | **17 / 18 (94.4)** | **IN** |  |
| Additional question on patient / parent questionnaire added by focus groups | - | Asking additional questions about pain eg. Where it is | N/A | N/A | N/A | N/A | **167 / 190 (88)** | **74 / 95 (78)** | No vote – discussion only | OUT | Not included as thought to be part of standard care |
| Global assessment by parent / patient  (continued) | Childhood health assessment questionnaire (CHAQ) or other functional assessment | *Childhood health assessment questionnaire (CHAQ) or other functional assessment* | 80 / 169 (47) | **132 / 169 (78)** | 63 / 136 (46) | **125 / 137 (91)** | Asking you or your child to complete a questionnaire that asks how easy or difficult it is for them to do things like get dressed, have a bath, do activities 118 / 193 (61) | Asking you to complete a list of questions that look at how easy or difficult it is for you to do things like get dressed, have a bath, do activities  52 /102 (51) | 3 / 18 (16.7)  Re-vote not specifying CHAQ:  **18 / 18 (100)** | **IN** | Changed to ‘age appropriate measure of activity’ (not specifying CHAQ as a tool) |
|  | Quality of life (QoL) tool | *Quality of life (QoL) tool* | 53 / 149 (36) | 96 / 149 (64) | 39 / 125 (31) | **102 / 125 (82)** | Asking your child how they feel emotionally in relation to JDM (questions relating to quality of life of mood)  131 / 196 (67) | Asking about your feelings in relation to your JDM  51 / 103 (50) | **18 / 18 (100)** | **IN** | Changed to ‘age appropriate measure of participation’ |
| Additional questions on patient / parent questionnaire added by focus groups | - | Asking how many days of school / college  missed due to JDM | N/A | N/A | N/A | N/A | 110 / 193 (57) | 41 / 101 (41) | No vote – discussion only | OUT | Thought to be part of standard care |
|  | - | Asking more questions about school – how things are / keeping up with peers | N/A | N/A | N/A | N/A | 116 / 191 (61) | 43 / 96  (45) | No vote – discussion only | OUT | Thought to be part of standard care |
| **Investigations** | Elevation of muscle enzymes | Elevation of muscle enzymes | **154 / 172 (90)** | **152 / 169 (90)** | **133 / 137 (97)** | **133 / 137 (97)** | Taking blood tests to monitor how active disease is  **177 / 197 (90)** | Taking blood tests to monitor how active disease is  **81 / 101 (80)** | **18 / 18 (100)** | **IN** |  |
|  | *CPK* | *Creatine phosphokinase (CK/CPK) above normal range* | **152 / 171 (89)** | **152 / 169 (90)** | **129 / 137 (94)** | **132 / 137 (96)** |  |  | Not voted on as an individual items | **IN** | Combined as one outcome (elevation of muscle enzymes) with drop down box to specify which enzymes elevated |
|  | *LDH* | *Lactate dehydrogenase (LDH/LD) above normal range* | **120 / 170 (71)** | **132 / 168 (79)** | **110 / 137 (80)** | **122 / 137 (89)** |  |  | Not voted on as an individual item | **IN** |  |
|  | *Aldolase* | *Aldolase above normal range* | 105 / 163 (64) | **118 / 161 (73)** | **96 / 130 (74)** | **108 / 130 (83)** |  |  | Not voted on as an individual item | **IN** |  |
|  | *AST / SGOT* | *AST/SGOT above normal range* | **120 / 169 (71)** | **128 / 169 (76)** | **112 / 136 (82)** | **120 / 137 (88)** |  |  | Not voted on as an individual item | **IN** |  |
|  | *ALT / SGPT* | *ALT/SGPT above normal range* | 108 / 168 (64) | **125 / 168 (74)** | **107 / 136 (79)** | **119 / 136 (88)** |  |  | Not voted on as an individual  item | **IN** |  |
|  | - | How important do you rate storing specimens for other biomarkers? | 39 / 167 (25) | **116 / 162 (72)** | 34 / 134 (25) | **117 / 136 (86)** | N/A | N/A | **18 / 18 (100)** | **IN*** | *Merged into one question at first and every visit asking ‘Has this patient had specimens taken that may be available for research projects? This may include DNA, serum, biomarkers, biopsy tissue to other material (asked to specify which specimens available if answer to this question is yes). |
|  | *-* | *How important do you rate storing DNA at diagnosis?* | 32 / 150 (21) | 100 / 146 (68) | 19 / 133 (14) | **105 / 135 (78)** | N/A | N/A | 0 (0)  Re-vote to combine in drop-down box = **18 / 18 (100)** | **IN** |  |
|  | *-* | *How important do you rate storing serum at diagnosis?* | 34 / 149 (23) | **111 / 145 (77)** | 19 / 133 (14) | **115 / 135 (85)** | N/A | N/A | 1 (5.6)  Re-vote to combine in drop-down box = **18 / 18 (100)** | **IN** |  |
|  | *-* | *Any other material you think should be stored at diagnosis?* | 20 / 110 (18) | 58 / 105 (55) | 14 / 99 (14) | 68 / 99 (69) | N/A | N/A | 0 (0)  Re-vote to combine in drop-down box = **18 / 18 (100)** | **IN** |  |
|  | Abnormal investigations since last review | Abnormal investigations since last review | 100 / 169 (59) | **121 / 164 (74)** | **103 / 136 (76)** | **122 / 135 (90)** | N/A | N/A | 7 / 18  (38.8) | OUT |  |
|  | *MRI* | *Abnormal MRI since last visit* | 84 / 153 (55) | **105 / 149 (70)** | 83 / 135 (61) | **112 / 135 (83)** | Asking your child to have a scan such as an MRI scan of their muscles to see how active the disease is  **132 / 188 (70)** | Asking you to have a scan such as an MRI scan of your muscles  **70 / 98 (71)** | 3 (16.6) | OUT |  |
|  | *Muscle biopsy* | *Abnormal biopsy since last visit* | 55 / 155 (35) | 83 **/** 151 (55) | 49 / 136 (36) | 92 / 136 (68) | N/A | N/A | 0 / 18 (0) | OUT |  |
|  | *EMG* | *Abnormal EMG since last visit* | 45 / 151 (30) | 70 / 148 (47) | 31 / 136 (23) | 75 / 136 (55) | N/A | N/A | 0 / 18 (0) | OUT |  |
|  | *Lung function tests* | *Abnormal lung function tests / X-ray / CT suggesting lung disease due to myositis* | 91 / 151 (60) | **112 / 150 (75)** | 93 / 137 (68) | **115 / 136 (85)** | N/A | N/A | 1 / 18 (5.6) | OUT |  |
|  | *ECG / ECHO* | *Abnormal electrocardiography (ECG) / echocardiogram suggesting cardiac disease* | 88 / 151 (58) | 100 / 149 (67) | 91 / 136 (67) | **106 / 136 (78)** | N/A | N/A | 0 / 18 (0) | OUT | Not commonly used in JDM. Difficult to define changes of myositis. |
| **Treatment** | Date treatment started | Date treatment started | **146 / 168 (87)** | **154 / 168 (92)** | **133 / 138 (96)** | **134 / 138 (97)** | Asking you about the medicines your child is taking at the moment  **172 / 197 (87)** | Asking you about the medicines you are taking at the moment  **78 / 100 (78)** | 0 / 18 (0) | OUT |  |
|  | Patient taking glucocorticoids | Patient taking glucocorticoids | **164 / 168 (98)** | **163 / 168 (97)** | **134 / 138 (99)** | **135 / 138 (98)** |  |  | 0 / 18 (0)  Re-vote: any drug **18 / 18 (100)** | **IN*** | *Combined into one outcome for ‘what treatment is the patient taking at present’ |
|  | *Oral* | *Oral glucocorticoids* | **163 / 169 (96)** | **162 / 168 (96)** | **137 / 138 (99)** | **134 / 138 (97)** |  |  | Not re-voted as individual item. From previous,  vote to combine in drop-down box = **18 / 18 (100)** | **IN** |  |
|  | *Intravenous* | *Intravenous glucocorticoids* | **159 / 168 (95)** | **160 / 167 (96)** | **135 / 138 (98)** | **132 / 138 (96)** |  |  |  |  |  |
|  | *Intramuscular* | *Intramuscular glucocorticoids* | **113 / 161 (70)** | **125 / 163 (77)** | **112 / 131 (85)** | **117 / 133 (88)** |  |  |  |  |  |
|  | *-* | *Intra-articular glucocorticoids* | 83 / 164 (51) | 104 / 164 (63) | 94 / 137 (69) | **103 / 137 (75)** |  |  | 1 / 18 (5.6) | OUT |  |
|  | *Topical* | *Topical glucocorticoids* | 78 / 168 (46) | 93 / 167 (56) | 81 / 137 (59) | **101 / 137 (74)** |  |  | 2 / 17 (11.1) | OUT |  |
|  | Other drugs (includes synthetic and biologic DMARDS (if yes: 16 tick box options to define names of drugs) | Patient taking a disease modifying anti-rheumatic drug? (if yes – to state which DMARDS) | **161 / 167 (96)** | **162 / 169 (96)** | **135 / 138 (98)** | **132 / 138 (96)** |  |  | Not voted on as individual item. Any drug **18 / 18 (100).** Include route **18 / 18 (100)**, Dose **16 / 18 (88)** and Frequency **17 / 18 (94)** | IN – combined with steroid options above and including route, dose and frequency of dosage | Included in outcome above ‘what treatment is patient taking at present’ – tick all that apply and for each drug include route, dose and frequency. |
|  |  | Patient taking a biologic? | **158 / 164 (96)** | **160 / 166 (96)** | **132 / 135 (98)** | **134 / 138 (97)** |  |  |  |  |  |
| Additional question asked to parents / patients – added by focus groups |  | Specific detail about medicines eg. how they make the child feel / any unwanted effects? | N/A | N/A | N/A | N/A | **158 / 194 (81)** | **68 / 94 (72)** | 1 / 18 (5.6) | OUT | Part of standard care |
| Treatment (continued) | Physiotherapy | Patient having physiotherapy / occupational therapy? | 105 / 169 (62) | 111 / 168 (66) | **110 / 138 (80)** | **112 / 138 (81)** | Asking you if your child is doing any physio-therapy exercises  128 / 193 (66) | Asking you if you are doing your physio-therapy exercises  52 / 100 (52) | **18 / 18 (100)** on re-vote for modified questions | **IN** | Modified to 2 questions on exercise routine to improve range of movement / muscle strength |
| Additional vote at consensus meeting | - | Should damage items be assessed? | N/A | N/A | N/A | N/A | N/A | N/A | **18 /18 (100)** | **IN** | Defined further post meeting, resulting in 54 damage outcomes |
| Voting post meeting via Survey monkey |  | Damage to be assessed annually | N/A | N/A | N/A | N/A | N/A | N/A | **18 / 18 (100)** | **IN** | Incorporated into instructions for form completion – not specific outcomes |
|  |  | Damage to be defined as persistent change, present for > 6 months, not responding to immune-suppressant therapy and not part of active inflammation | N/A | N/A | N/A | N/A | N/A | N/A | **17 / 18 (94.4)** | **IN** |  |
|  |  | All sections of Myositis Damage Index (MDI) to be included in damage section (including peripheral vascular damage) | N/A | N/A | N/A | N/A | N/A | N/A | **17 / 18 (94.4)** on 2^nd^ damage survey monkey | **IN** | Consequence of this decision = 11 domains plus a global damage VAS |
|  |  | Exclude items in italics on MDI | N/A | N/A | N/A | N/A | N/A | N/A | **15 / 18 (83.3)** | **IN** |  |
|  |  | Keep items relevant to adults only in the damage section (making clear that only for adults) | N/A | N/A | N/A | N/A | N/A | N/A | **17 / 18 (94.4)** | **IN** |  |
| Extra questions considered at consensus meeting raised from Delhi / patient & parent survey as additional suggestions from participants |  | Country patient residing in (to understand local environment) | N/A | N/A | N/A | N/A | N/A | N/A | 0 (0) | OUT |  |
|  |  | Socioeconomic status | N/A | N/A | N/A | N/A | N/A | N/A | 0 (0) | OUT |  |
|  |  | Zip code | N/A | N/A | N/A | N/A | N/A | N/A | 11 / 14 (78) on 2^nd^ vote | OUT |  |
|  |  | History of drug intake | N/A | N/A | N/A | N/A | N/A | N/A | 0 (0) | OUT |  |
|  |  | History of toxic substance exposure | N/A | N/A | N/A | N/A | N/A | N/A | 0 (0) | OUT |  |
|  |  | History of other disease | N/A | N/A | N/A | N/A | N/A | N/A | 0 (0) | OUT |  |
|  |  | Exposure to infectious contacts prior to diagnosis | N/A | N/A | N/A | N/A | N/A | N/A | 0 (0) | OUT |  |
|  |  | Any proceeding exposure | N/A | N/A | N/A | N/A | N/A | N/A | 0 (0) | OUT |  |
|  |  | Diet before diagnosis | N/A | N/A | N/A | N/A | N/A | N/A | 0 (0) | OUT |  |
|  |  | Type of season / flare | N/A | N/A | N/A | N/A | N/A | N/A | 0 (0) | OUT |  |
|  |  | Muscle cramps | N/A | N/A | N/A | N/A | N/A | N/A | 0 (0) | OUT |  |
|  |  | Muscle ultrasound | N/A | N/A | N/A | N/A | N/A | N/A | 0 (0) | OUT |  |
|  |  | Swallow test | N/A | N/A | N/A | N/A | N/A | N/A | 0 (0) | OUT |  |
|  |  | IgE measurement | N/A | N/A | N/A | N/A | N/A | N/A | 0 (0) | OUT |  |
|  |  | ANCA measurement | N/A | N/A | N/A | N/A | N/A | N/A | 0 (0) | OUT |  |
|  |  | Abdominal ultrasound | N/A | N/A | N/A | N/A | N/A | N/A | 0 (0) | OUT |  |
|  |  | Swallow test | N/A | N/A | N/A | N/A | N/A | N/A | 0 (0) | OUT |  |
|  |  | Pulmonary function tests | N/A | N/A | N/A | N/A | N/A | N/A | 0 (0) | OUT |  |
|  |  | Reproductive status | N/A | N/A | N/A | N/A | N/A | N/A | 0 (0) | OUT |  |
|  |  | Walking distance | N/A | N/A | N/A | N/A | N/A | N/A | 0 (0) | OUT |  |
|  |  | Signs of damage | N/A | N/A | N/A | N/A | N/A | N/A | 0 (0) | OUT |  |
|  |  | Grading of arthritis | N/A | N/A | N/A | N/A | N/A | N/A | 0 (0) | OUT |  |

**Supplementary Table S2: Patients opinions on outcome measures - results of UK Young Person’s JDM focus group discussion (n=6, aged 15-22 years).**

|  | **Patient Reported Outcome Measure (PROM):** | **Patients’ opinion on PROM – summary from focus group** |
| --- | --- | --- |
| 1 | JDMAR (Juvenile Dermatomyositis Multidimensional Assessment Report)[23]: | A firm favourite. Could relate to the questions, which were targeted and relevant to JDM. Particularly like the medication section and the fact it included symptoms of JDM. |
| 2 | Childhood Health Assessment Questionnaire (CHAQ)[22]: | Not liked by this age group. Recognised that it may be relevant to younger children but thought questions were patronising, not useful and focused on negative issues. |
| 3 | Patient global Visual Analogue Scale (VAS)[22]: | Found a score of 0-100 mm too big; preferred 0-10 cm. Overall disliked stating a particular point on a scale. |
| 4 | Child Health Questionnaire (CHQ)[22,24]: | Liked some sections (eg. Interaction with family members and questions on self esteem) but not others (eg. Physical activities or disease related to peers). Overall felt to be relevant up to the age of 17 years but not applicable for older age groups. |
| 5 | Short Form-36 (SF 36)[22]: | Liked by this age group but agreed not applicable for younger children. |
| 6 | Pediatric Quality of Life Inventory (PedsQL) [24,27]: | Thought to be good for younger children and applicable up to age of 18 years. Relevant and particularly liked questions relating to school. Felt it was difficult to assess emotions over 1 month due to range of emotions experienced in this time, and thought important to state context (eg. Stressed due to exams versus disease flare). |
| 7 | Quality of My Life Questionnaire (QoML)[24,27]: | Overall liked, provided health professionals used it to ask questions about a patients score. Negative points were that health and quality of life are intertwined and cannot be easily separated. Felt to be slightly vague. |
| 8 | EuroQoL-5 dimension (EQ-5D)[26]: | Thought important to have child and parent opinions, depending on how young child is. Preferred the activities section in this questionnaire compared to other questionnaires as felt that the activities could be adapted to their own lives (better than CHQ). |
| 9 | Children’s Dermatology Life Quality Index (DLQI)[22]: | Thought that some questions were relevant and could be added to the JDMAR – especially how embarrassed / self conscious have you been because of your skin. Recognised that skin disease can lower self-esteem. |
| 10 | DISABKIDS Chronic Generic Measure[24]: | Not discussed (not able to access questionnaire due to licensing/ registration requirements). |

**Supplementary Table S3: Data feasibility questionnaire for testing the dataset in practice:**

| 1. **Questions about the dataset** | | 1. **Questions about you** |
| --- | --- | --- |
| 1 | Are there any questions that you think are unclear or not understandable?  Yes. If yes, which ones  No | Your name  ________________________________________________________ |
| 2 | Are there any definitions of variables that you think are unclear / inaccurate / not understandable?  Yes*  No  Not applicable (did not need to look at definitions)  *If yes, which ones: ____________________________________________ | Were you present at the consensus meeting in March 2015 OR are these data entry forms new to you?  I was present at the meeting  These data entry forms are new to me  I was not present at the meeting but I did complete the Delphi survey in 2015 and therefore have seen some of the variables before |
| 3 | Are there any variables in the dataset that you are unable to complete in your clinical practice?  Yes*  No  *If yes, which ones, and why are you unable to complete them? _______________________________________________________________________ | Your current primary role:  Rheumatologist (specialist paediatric / adult physician)  Paediatrician or physician with an interest in rheumatology  Dermatologist  Neurologist  Academic / researcher in basic science / rheumatology / immunology / other  Trainee (please state specialty) ____________  Therapist  Other: Please state __________ |
| 4 | Are there any questions that you think are repeated / overlap that could be combined or removed?  Yes*  No  *If yes, which ones: _____________________________________________ | Experience in specialty (tick which option applies):  Consultant / academic / Allied Health Professional with ≥ 10 years experience  Consultant / academic / Allied Health Professional with 5-9 years experience  Consultant / academic / Allied Health Professional with < 5 years experience  Trainee  Other (please state)___________________ |
| 5 | Are there any questions that you would like to see removed from the dataset?  Yes*  No  * If ‘yes’ list questions & reasons why you think they should be removed: ____________________________________________________ | How many paediatric patients with JDM or other Idiopathic Inflammatory Myopathies are directly under your care / do you regularly review per year? (Choose one option)  <5  5-9  10-19  20-29  30+ |
| 6 | Is this dataset clinically helpful to you?  Yes  No  Comments:_____________________________________________________ | Area of practice:  Europe  USA / Canada  Latin America  Asia  Africa  Australia / Oceania  Middle East  Other (state) _____________ |
| 7 | How long did it take you to complete data entry for the dataset for each patient?  minutes  Number of patients tested | Membership of special interest groups (tick all that apply)  IMACS  CARRA  PRINTO  UK JDRG  PReS JDM group  MyoNet (previously EuMyoNet)  Euromyositis  Other (please state) ___________________ |
| 8 | Any other comments / suggestions? |  |

**Supplementary Table S4: Accessibility of investigations to participants in area of practice according to responses received in Delphi 1:**

| **Investigation** | **Easily accessible: number (%) of responses** | **Not easily accessible: number (%) of responses** | **Unable to comment: number (%) of responses** |
| --- | --- | --- | --- |
| Muscle biopsy | 169 (78.6%) | 38 (17.7) | 8 (3.7) |
| Muscle enzymes | 212 (98.6) | 1 (0.5) | 2 (0.9) |
| Creatine phosphokinase (CPK) | 211 (99.5) | 0 (0) | 0 (0) |
| Lactate dehydrogenase (LDH) | 208 (98.1) | 2 (0.9) | 1 (0.5) |
| Aldolase | 147 (69.7) | 61 (28.9) | 3 (1.4) |
| Aspartate Aminotransferase (AST) / Serum Glutamic Oxaloacetic Transaminase (SGOT) | 209 (99.1) | 2 (0.9) | 0 (0) |
| Alanine transaminase (ALT) / Serum glutamic-pyruvic transaminase (SGPT) | 210 (99.5) | 1 (0.5) | 0 (0) |
| Electromyography (EMG) | 169 (78.6) | 42 (19.5) | 4 (1.9) |
| Magnetic Resonance Imaging (MRI) | 188 (87.4) | 25 (11.6) | 2 (0.9) |
| Antinuclear antibody (ANA) | 210 (97.7) | 4 (1.9) | 1 (0.5) |
| Myositis Specific Antibodies (MSA) | 129 (60) | 77 (35.8) | 9 (4.2) |
| Myositis Associated Antibodies (MAA) | 150 (69.8) | 59 (27.4) | 6 (2.8) |
| Storing specimens | 125 (58.1) | 62 (28.8) | 28 (13) |
| Storing Deoxyribonucleic acid (DNA) | 106 (52.2) | 70 (34.5) | 25 (12.3) |
| Storing serum | 128 (63.4) | 47 (23.3) | 26 (12.9) |
| Storing other material | 78 (38.6) | 54 (26.7) | 69 (34.1) |

Supplementary Tables S5: Dataset forms

| **FORM A – COMPLETE AT FIRST / BASELINE VISIT ONLY (or first data entry) - Please also complete Form B and C**  **[Full glossary of definitions available to aid completion]** | | | | |
| --- | --- | --- | --- | --- |
| **Category** | **Item** | **Data entry options on database** | **Additional data entry options on database / conditional items if answer to preceding question is ‘yes’** | |
| **Demographics** | Date of birth: year & month of birth +/- day of birth. (Estimate date of birth if not known by patient) | YYYY / MM / [DD]  []  [day optional] |  | |
|  | Sex of patient | Male Female |  | |
| **Diagnostic factors** | Date (year & month) of first symptom of myositis | YYYY / MM |  | |
|  | Date (year & month) of diagnosis of JDM | YYYY / MM |  | |
|  | At the time of diagnosis, did the patient have proximal muscle weakness | Yes  No  Unknown |  | |
|  | At the time of diagnosis, did the patient have typical skin features of JDM (Gottron’s / Heliotrope)? | Yes  No  Unknown |  | |
|  | Was an MRI scan done at diagnosis? | Yes *  No  Unknown | *** If yes, was the result:**  Normal  Abnormal consistent with myositis  Abnormal but not consistent with myositis  Not available | |
|  | Was a muscle biopsy done at diagnosis? | Yes *  No  Unknown | *** If yes, was the result:**  Normal*  Abnormal consistent with myositis*  Abnormal but not consistent with myositis*  Not available | |
|  |  |  | ***Muscle biopsy tool data if available:**  **Overall impression of abnormality** **VAS** 0-10 (scored by pathologist) .  Inflammatory domain (0-12)  Muscle fibre domain (0-10)  Vascular domain (0-3)  Connective tissue domain (0-2) | |
|  | Were Myositis Specific Antibodies (MSA) tested at diagnosis? | Yes *  No  Unknown | *** If yes, was any MSA positive?**  Yes *  No  Not available | ***Which MSA was positive?**  TIF-1γ / (anti-p155/p140)  NXP-2 (anti-MJ)  Anti-MDA5  Anti synthetase (e.g.Anti-Jo-1)  Anti Mi-2  Anti SAE  Anti-SRP  Other ________ |
| **Diagnostic factors (continued)** | Were Myositis Associated Antibodies (MAA) tested at diagnosis? | Yes *  No  Unknown | *** If yes, were any MSA positive?**  Yes *  No  Not available | ***If yes, which MAA were positive?**  Anti-Ro  Anti-La  Anti-U1-RNP  Anti-PM-Scl  Anti-Sm  Anti-Ku  Other U-RNP  Anti-Topo  Other_________ |
| **Treatments received prior to diagnosis of JDM** | Did this patient receive systemic glucocorticoid prior to diagnosis of JDM? | Yes*  No  Unknown | ***If yes, tick which apply:**  Oral corticosteroids  Intravenous corticosteroids  Intramuscular corticosteroids | |
|  | Did this patient receive any synthetic or biologic disease modifying anti-rheumatic drug (DMARD) prior to the diagnosis of JDM? | Yes*  No  Unknown | *If yes, tick ALL that apply:  Methotrexate  Ciclosporin A  Azathioprine  Hydroxychloroquine  IV Immunoglobulin  Mycophenolate Mofetil  Cyclophosphamide  Rituximab  Infliximab  Adalimumab  Etanercept  Plasmapheresis  Other ________ | |
| **PLEASE ALSO COMPLETE FORM B FOR EVERY VISIT & FORM C AT BASELINE AND ANNUAL VISITS** | | | | |

| **FORM B – COMPLETE AT BASELINE AND EVERY VISIT [Since time of last data completion]**  **[Full glossary of definitions available to aid completion and muscle strength testing forms for MMT8 and CMAS]** | | | |
| --- | --- | --- | --- |
| **Category** | **Item** | **Data entry options on database** | **Additional data entry options on database / conditional items if answer to preceding question is ‘yes’** |
| **Growth** | Height of patient (in centimetres) | . cm  Not measured / unknown |  |
|  | Weight of patient (in kilograms) | . kg  Not measured / unknown |  |
| **Muscular involvement** | Presence of symmetric proximal muscle weakness | Yes  No  Unknown |  |
|  | Childhood myositis assessment scale (CMAS) score completed | Yes  No  Unknown | CMAS score:  / 52 |
|  | Manual Muscle Testing (MMT8) score completed | Yes  No  Unknown | MMT8 score:  / 80 |
|  | **Visual Analogue Scale (VAS) score for global muscle disease activity**  (Measurement to 0.1cm allowed) | VAS measured  Yes*  No  Unknown | * At this time, overall disease activity **for muscle disease** is:  ☐ ☐ ☐ ☐ ☐ ☐ ☐ ☐ ☐ ☐ ☐ ☐ ☐ ☐ ☐ ☐ ☐ ☐ ☐ ☐ ☐  0 0.5 1 1.5 2 2.5 3 3.5 4 4.5 5 5.5 6 6.5 7 7.5 8 8.5 9 9.5 10  Not Very  Active Active |
| **Skeletal involvement** | Arthritis due to myositis | Yes  No  Unknown |  |
|  | Joint contractures due to myositis | Yes  No  Unknown |  |
|  | **Visual Analogue Scale (VAS) score for global skeletal disease activity**  (Measurement to 0.1cm allowed) | VAS measured  Yes*  No  Unknown | * At this time, overall disease activity **for skeletal disease** is:  ☐ ☐ ☐ ☐ ☐ ☐ ☐ ☐ ☐ ☐ ☐ ☐ ☐ ☐ ☐ ☐ ☐ ☐ ☐ ☐ ☐  0 0.5 1 1.5 2 2.5 3 3.5 4 4.5 5 5.5 6 6.5 7 7.5 8 8.5 9 9.5 10  Not Very  Active Active |
| **Cutaneous involvement** | Gottron’s papules or Gottron’s sign | Yes  No  Unknown |  |
| **Cutaneous involvement**  (continued) | Heliotrope rash | Yes  No  Unknown |  |
|  | Periungual capillary loop changes (plus measure of capillary density if available) | Yes  No  Unknown |  |
|  | Malar or facial erythema | Yes  No  Unknown |  |
|  | Linear extensor erythema | Yes  No  Unknown |  |
|  | ‘V’ sign | Yes  No  Unknown |  |
|  | Shawl sign | Yes  No  Unknown |  |
|  | Non-sun-exposed erythema | Yes  No  Unknown |  |
|  | Extensive cutaneous erythema, which may include erythroderma. | Yes  No  Unknown |  |
|  | Livedo reticularis | Yes  No  Unknown |  |
|  | Cutaneous ulceration | Yes  No  Unknown |  |
|  | Mucus membrane lesions | Yes  No  Unknown |  |
|  | Mechanics hand’s | Yes  No  Unknown |  |
|  | Cuticular overgrowth | Yes  No  Unknown |  |
| Cutaneous involvement (continued) | Subcutaneous oedema | Yes  No  Unknown |  |
|  | Panniculitis | Yes  No  Unknown |  |
|  | Alopecia (non-scarring) | Yes  No  Unknown |  |
|  | Calcinosis (associated with active disease) | Yes  No  Unknown |  |
|  | **Visual Analogue Scale (VAS) score for global cutaneous disease activity**  (Measurement to 0.1cm allowed) | VAS measured  Yes*  No  Unknown | * At this time, overall disease activity **for cutaneous disease** is:  ☐ ☐ ☐ ☐ ☐ ☐ ☐ ☐ ☐ ☐ ☐ ☐ ☐ ☐ ☐ ☐ ☐ ☐ ☐ ☐ ☐  0 0.5 1 1.5 2 2.5 3 3.5 4 4.5 5 5.5 6 6.5 7 7.5 8 8.5 9 9.5 10  Not Very  Active Active |
| **Features suggestive of myositis overlap** | Does this patient have a myositis overlap condition? | Yes *  No  Unknown | ***Overlap features:**  Scleroderma  Lupus  Mixed Connective Tissue Disease (RNP+ve)  Other _____________ |
|  | Raynaud’s phenomenon | Yes  No  Unknown |  |
|  | Sclerodactyly | Yes  No  Unknown |  |
| **Gastro-intestinal involvement** | Dysphagia due to myositis | Yes  No  Unknown |  |
|  | Abdominal pain due to myositis | Yes  No  Unknown |  |
| **Gastro-intestinal involvement**  (continued) | Gastro-intestinal ulceration due to myositis | Yes  No  Unknown |  |
|  | **Visual Analogue Scale (VAS) score for global gastrointestinal disease activity**  (Measurement to 0.1cm allowed) | VAS measured  Yes*  No  Unknown | * At this time, overall disease activity **for gastrointestinal disease** is:  ☐ ☐ ☐ ☐ ☐ ☐ ☐ ☐ ☐ ☐ ☐ ☐ ☐ ☐ ☐ ☐ ☐ ☐ ☐ ☐ ☐  0 0.5 1 1.5 2 2.5 3 3.5 4 4.5 5 5.5 6 6.5 7 7.5 8 8.5 9 9.5 10  Not Very  Active Active |
| **Pulmonary involvement** | Pulmonary involvement / respiratory muscle weakness or interstitial lung disease due to myositis | Yes  No  Unknown |  |
|  | Dysphonia due to myositis | Yes  No  Unknown |  |
|  | **Visual Analogue Scale (VAS) score for global pulmonary disease activity**  (Measurement to 0.1cm allowed) | VAS measured  Yes*  No  Unknown | * At this time, overall disease activity **for pulmonary disease** is:  ☐ ☐ ☐ ☐ ☐ ☐ ☐ ☐ ☐ ☐ ☐ ☐ ☐ ☐ ☐ ☐ ☐ ☐ ☐ ☐ ☐  0 0.5 1 1.5 2 2.5 3 3.5 4 4.5 5 5.5 6 6.5 7 7.5 8 8.5 9 9.5 10  Not Very  Active Active |
| **Cardiovascular involvement** | Cardiovascular involvement due to myositis | Yes  No  Unknown |  |
|  | Blood pressure (BP) recording | BP measured  Yes*  No  Unknown | *  /  (systolic / diastolic) |
|  | Blood pressure elevated suggesting hypertension (for age of patient) | Yes  No  Unknown |  |
|  | **Visual Analogue Scale (VAS) score for global cardiovascular disease activity**  (Measurement to 0.1cm allowed) | VAS measured  Yes*  No  Unknown | * At this time, overall disease activity **for cardiovascular disease** is:  ☐ ☐ ☐ ☐ ☐ ☐ ☐ ☐ ☐ ☐ ☐ ☐ ☐ ☐ ☐ ☐ ☐ ☐ ☐ ☐ ☐  0 0.5 1 1.5 2 2.5 3 3.5 4 4.5 5 5.5 6 6.5 7 7.5 8 8.5 9 9.5 10  Not Very  Active Active |
| **Constitutional features** | Fever (>38^o^C) due to myositis | Yes  No  Unknown |  |
|  | Weight loss (>5%) due to myositis | Yes  No  Unknown |  |
|  | Fatigue due to myositis | Yes  No  Unknown |  |
|  | **Visual Analogue Scale (VAS) score for global constitutional disease activity**  (Measurement to 0.1cm allowed) | VAS measured  Yes*  No  Unknown | * At this time, overall disease activity **for constitutional features** is:  ☐ ☐ ☐ ☐ ☐ ☐ ☐ ☐ ☐ ☐ ☐ ☐ ☐ ☐ ☐ ☐ ☐ ☐ ☐ ☐ ☐  0 0.5 1 1.5 2 2.5 3 3.5 4 4.5 5 5.5 6 6.5 7 7.5 8 8.5 9 9.5 10  Not Very  Active Active |
| **Global disease assessment by clinician** | Physician Visual Analogue Scale (VAS) score of global disease activity  (Measurement to 0.1cm allowed) | VAS measured  Yes*  No  Unknown | * At this time, overall **global disease activity** is:  ☐ ☐ ☐ ☐ ☐ ☐ ☐ ☐ ☐ ☐ ☐ ☐ ☐ ☐ ☐ ☐ ☐ ☐ ☐ ☐ ☐  0 0.5 1 1.5 2 2.5 3 3.5 4 4.5 5 5.5 6 6.5 7 7.5 8 8.5 9 9.5 10  Not Very  Active Active |
|  | Physician Visual Analogue Scale (VAS) score of global extra-muscular disease activity  (Measurement to 0.1cm allowed) | VAS measured  Yes*  No  Unknown | * At this time, overall disease activity **for** **extra-muscular disease** is:  ☐ ☐ ☐ ☐ ☐ ☐ ☐ ☐ ☐ ☐ ☐ ☐ ☐ ☐ ☐ ☐ ☐ ☐ ☐ ☐ ☐  0 0.5 1 1.5 2 2.5 3 3.5 4 4.5 5 5.5 6 6.5 7 7.5 8 8.5 9 9.5 10  Not Very  Active Active |
| **Global disease assessment by patient / parent** | Patient / parent Visual Analogue Scale (VAS) score for global disease activity  (Measurement to 0.1cm allowed) | VAS measured  Yes*  No  Unknown | * At this time, I am / my child is:  ☐ ☐ ☐ ☐ ☐ ☐ ☐ ☐ ☐ ☐ ☐ ☐ ☐ ☐ ☐ ☐ ☐ ☐ ☐ ☐ ☐  0 0.5 1 1.5 2 2.5 3 3.5 4 4.5 5 5.5 6 6.5 7 7.5 8 8.5 9 9.5 10  very very  well poorly  Who completed the form?  Mother  / Father  / Other carer  / Patient  / Unknown |
|  | Patient / parent Visual Analogue Scale (VAS) score for pain  (Measurement to 0.1cm allowed) | VAS measured  Yes*  No  Unknown | * At this time, my pain is / my child’s pain is:  ☐ ☐ ☐ ☐ ☐ ☐ ☐ ☐ ☐ ☐ ☐ ☐ ☐ ☐ ☐ ☐ ☐ ☐ ☐ ☐ ☐  0 0.5 1 1.5 2 2.5 3 3.5 4 4.5 5 5.5 6 6.5 7 7.5 8 8.5 9 9.5 10  No Very  Pain severe pain |
| **Patient / parent reported outcome measure (PROM)** | Use of an age-appropriate patient / parent reported measure (PROM) of function | Yes*  No  Unknown / not measured | **Optional data entry**:  *PROM used  *Score . |
|  | Use of an age-appropriate patient / parent reported measure (PROM) of quality of life | Yes*  No  Unknown / not measured | **Optional data entry**:  *PROM used  *Score |
| **Investigations** | Elevation of any muscle enzyme (including CPK, LDH, aldolase, AST / SGOT, ALT / SGPT) above normal range | Yes*  No  Unknown | **Enzyme elevated:**  CPK  LDH  Aldolase  AST / SGOT  ALT / SGPT |
| **Specimens available** | Has this patient had specimens taken that may be available for specific research projects? This may include DNA, serum, biomarkers, biopsy tissue or other material. | Yes*  No  Unknown | **If yes:**  DNA  Serum  Other samples / tissue______________ |
| **Treatment** | Is the patient on treatment (now or since last visit)? | Yes*  No  Unknown   \| *** If yes, tick all that apply** \| **Dose** \| **Route** \| **Frequency of administration** \| \| --- \| --- \| --- \| --- \| \| Oral corticosteroids \|  \|  \|  \| \| Intravenous (IV) corticosteroids \|  \|  \|  \| \| Intramuscular (IM) corticosteroids \|  \|  \|  \| \| Methotrexate \|  \|  \|  \| \| Ciclosporin A \|  \|  \|  \| \| Azathioprine \|  \|  \|  \| \| Hydroxychloroquine \|  \|  \|  \| \| IV Immunoglobulin \|  \|  \|  \| \| Mycophenolate Mofetil \|  \|  \|  \| \| Cyclophosphamide \|  \|  \|  \| \| Rituximab \|  \|  \|  \| \| Infliximab \|  \|  \|  \| \| Adalimumab \|  \|  \|  \| \| Etanercept \|  \|  \|  \| \| Plasmapheresis \|  \|  \|  \| \| Other ___________ \|  \|  \|  \| | |
| **Treatment** (continued) | Is the patient doing a regular exercise routine prescribed by a healthcare professional aimed at: | **Improving / maintaining**  **A) range of movement:**  Yes  No  Unknown  **B) muscle strength:**  Yes  No  Unknown |  |
| **Please also complete form C where applicable** | | | |

| **Form C – COMPLETE AT BASELINE AND ONCE YEARLY THEREAFTER**  **[Full glossary of definitions available to aid completion]**  **Please note: To qualify as damage, items need to be present for > 6 months, not responding to immunosuppressive therapy and not part of active inflammation** | | | |
| --- | --- | --- | --- |
| **Category** | **Item** | **Data entry options on database** | **Additional data entry options on database** |
| **Muscular - damage items** (present for > 6 months) | Muscle atrophy (clinical) | Yes  No  Unknown |  |
|  | Muscle weakness not attributable to active muscle disease | Yes  No  Unknown |  |
|  | Muscle dysfunction: decrease in aerobic exercise capacity | Yes  No  Unknown |  |
|  | **Visual Analogue Scale (VAS) score for muscle disease damage**  (Measurement to 0.1cm allowed) | VAS measured  Yes*  No  Unknown | * At this time, overall disease damage **for muscle disease** is:  ☐ ☐ ☐ ☐ ☐ ☐ ☐ ☐ ☐ ☐ ☐ ☐ ☐ ☐ ☐ ☐ ☐ ☐ ☐ ☐ ☐  0 0.5 1 1.5 2 2.5 3 3.5 4 4.5 5 5.5 6 6.5 7 7.5 8 8.5 9 9.5 10  Absent Maximum |
| **Skeletal - damage items** (present for > 6 months) | Joint contractures (due to myositis) | Yes  No  Unknown |  |
|  | Osteoporosis with fracture or vertebral collapse (excluding avascular necrosis) | Yes  No  Unknown |  |
|  | Avascular necrosis | Yes  No  Unknown |  |
|  | Deforming arthropathy | Yes  No  Unknown |  |
|  | **Visual Analogue Scale (VAS) score for skeletal disease damage**  (Measurement to 0.1cm allowed) | VAS measured  Yes*  No  Unknown | * At this time, overall disease damage **for skeletal disease** is:  ☐ ☐ ☐ ☐ ☐ ☐ ☐ ☐ ☐ ☐ ☐ ☐ ☐ ☐ ☐ ☐ ☐ ☐ ☐ ☐ ☐  0 0.5 1 1.5 2 2.5 3 3.5 4 4.5 5 5.5 6 6.5 7 7.5 8 8.5 9 9.5 10  Absent Maximum |
| **Cutaneous - damage items** (present for > 6 months) | Calcinosis (persistent) | Yes  No  Unknown |  |
|  | Alopecia (scarring) | Yes  No  Unknown |  |
|  | Cutaneous scarring or atrophy (depressed scar or cutaneous atrophy) | Yes  No  Unknown |  |
|  | Poikiloderma | Yes  No  Unknown |  |
|  | Lipoatrophy / lipodystrophy | Yes  No  Unknown |  |
|  | **Visual Analogue Scale (VAS) score for cutaneous disease damage**  (Measurement to 0.1cm allowed) | VAS measured  Yes*  No  Unknown | * At this time, overall disease damage **for cutaneous disease** is:  ☐ ☐ ☐ ☐ ☐ ☐ ☐ ☐ ☐ ☐ ☐ ☐ ☐ ☐ ☐ ☐ ☐ ☐ ☐ ☐ ☐  0 0.5 1 1.5 2 2.5 3 3.5 4 4.5 5 5.5 6 6.5 7 7.5 8 8.5 9 9.5 10  Absent Maximum |
| **Gastro-intestinal - damage items** (present for > 6 months)  Gastro-intestinal damage items (continued) | Dysphagia (persistent) | Yes  No  Unknown |  |
|  | Gastrointestinal dysmotility, constipation, diarrhoea or abdominal pain (persistent) | Yes  No  Unknown |  |
|  | Infarction or resection of bowel or other gastrointestinal organs | Yes  No  Unknown |  |
|  | **Visual Analogue Scale (VAS) score for gastrointestinal disease damage**  (Measurement to 0.1cm allowed) | VAS measured  Yes*  No  Unknown | * At this time, overall disease damage **for gastrointestinal disease** is:  ☐ ☐ ☐ ☐ ☐ ☐ ☐ ☐ ☐ ☐ ☐ ☐ ☐ ☐ ☐ ☐ ☐ ☐ ☐ ☐ ☐  0 0.5 1 1.5 2 2.5 3 3.5 4 4.5 5 5.5 6 6.5 7 7.5 8 8.5 9 9.5 10  Absent Maximum |
| **Pulmonary - damage items** (present for > 6 months) | Dysphonia (persistent) | Yes  No  Unknown |  |
|  | Impaired lung function due to respiratory muscle damage | Yes  No  Unknown |  |
|  | Pulmonary fibrosis | Yes  No  Unknown |  |
|  | Pulmonary hypertension | Yes  No  Unknown |  |
|  | **Visual Analogue Scale (VAS) score for pulmonary disease damage**  (Measurement to 0.1cm allowed) | VAS measured  Yes*  No  Unknown | * At this time, overall disease damage **for pulmonary disease** is:  ☐ ☐ ☐ ☐ ☐ ☐ ☐ ☐ ☐ ☐ ☐ ☐ ☐ ☐ ☐ ☐ ☐ ☐ ☐ ☐ ☐  0 0.5 1 1.5 2 2.5 3 3.5 4 4.5 5 5.5 6 6.5 7 7.5 8 8.5 9 9.5 10  Absent Maximum |
| **Cardio-vascular damage items** (present for > 6 months)  Cardio-vascular damage items (continued) | Hypertension requiring treatment for > 6 months | Yes  No  Unknown |  |
|  | Ventricular dysfunction or cardiomyopathy | Yes  No  Unknown |  |
|  | Assessed in adults (> 18 years of age): Angina or coronary artery bypass [Can be documented in children < 18 years of age if applicable] | Yes  No  Unknown |  |
|  | Assessed in adults (> 18 years of age): Myocardial infarction [Can be documented in children < 18 years of age if applicable] | Yes  No  Unknown |  |
|  | **Visual Analogue Scale (VAS) score for cardiovascular disease damage**  (Measurement to 0.1cm allowed) | VAS measured  Yes*  No  Unknown | * At this time, overall disease damage **for cardiovascular disease** is:  ☐ ☐ ☐ ☐ ☐ ☐ ☐ ☐ ☐ ☐ ☐ ☐ ☐ ☐ ☐ ☐ ☐ ☐ ☐ ☐ ☐  0 0.5 1 1.5 2 2.5 3 3.5 4 4.5 5 5.5 6 6.5 7 7.5 8 8.5 9 9.5 10  Absent Maximum |
| **Peripheral vascular damage** (present for > 6 months) | Tissue or pulp loss | Yes  No  Unknown |  |
|  | Digit loss or limb loss or resection | Yes  No  Unknown |  |
|  | Venous or arterial thrombosis with swelling, ulceration or venous stasis | Yes  No  Unknown |  |
|  | Assessed in adults (>18 years of age): Claudication [Can be documented in children < 18 years of age if applicable] | Yes  No  Unknown |  |
|  | **Visual Analogue Scale (VAS) score for peripheral vascular disease damage**  (Measurement to 0.1cm allowed) | VAS measured  Yes*  No  Unknown | * At this time, overall disease damage **for peripheral vascular disease** is:  ☐ ☐ ☐ ☐ ☐ ☐ ☐ ☐ ☐ ☐ ☐ ☐ ☐ ☐ ☐ ☐ ☐ ☐ ☐ ☐ ☐  0 0.5 1 1.5 2 2.5 3 3.5 4 4.5 5 5.5 6 6.5 7 7.5 8 8.5 9 9.5 10  Absent Maximum |
| **Pubertal status** | Pubertal status of patient – defined by Tanner score [Self-assessment forms can be used] | Tanner Score measured  Yes *  No  Unknown | * Tanner score  1  2  3  4  5 |
| **Endocrine damage** (present for > 6 months) | Growth failure | Yes  No  Unknown |  |
|  | Delay in development of secondary sexual characteristics [>2 SD beyond mean for age] | Yes  No  Unknown |  |
|  | Hirsutism or hypertrichosis | Yes  No  Unknown |  |
|  | Irregular menses | Yes  No  Unknown |  |
|  | Primary or secondary amenorrhea | Yes  No  Unknown |  |
|  | Diabetes mellitus | Yes  No  Unknown |  |
|  | In adults (> 18 years of age): Infertility – male or female [Can be documented in children < 18 years of age if applicable] | Yes  No  Unknown |  |
|  | In adults (>18 years of age): Sexual dysfunction [Can be documented in children < 18 years of age if applicable] | Yes  No  Unknown |  |
|  | **Visual Analogue Scale (VAS) score for endocrine disease damage**  (Measurement to 0.1cm allowed) | VAS measured  Yes*  No  Unknown | * At this time, overall disease damage **for endocrine disease** is:  ☐ ☐ ☐ ☐ ☐ ☐ ☐ ☐ ☐ ☐ ☐ ☐ ☐ ☐ ☐ ☐ ☐ ☐ ☐ ☐ ☐  0 0.5 1 1.5 2 2.5 3 3.5 4 4.5 5 5.5 6 6.5 7 7.5 8 8.5 9 9.5 10  Absent Maximum |
| **Ocular - damage items** (present for > 6 months) | Cataract resulting in visual loss | Yes  No  Unknown |  |
|  | Visual loss, other, not secondary to cataract | Yes  No  Unknown |  |
|  | **Visual Analogue Scale (VAS) score for ocular disease damage**  (Measurement to 0.1cm allowed) | VAS measured  Yes*  No  Unknown | * At this time, overall disease damage **for ocular disease** is:  ☐ ☐ ☐ ☐ ☐ ☐ ☐ ☐ ☐ ☐ ☐ ☐ ☐ ☐ ☐ ☐ ☐ ☐ ☐ ☐ ☐  0 0.5 1 1.5 2 2.5 3 3.5 4 4.5 5 5.5 6 6.5 7 7.5 8 8.5 9 9.5 10  Absent Maximum |
| **Infection** (present for > 6 months) | Chronic infection | Yes  No  Unknown |  |
|  | Multiple infections | Yes  No  Unknown |  |
|  | **Visual Analogue Scale (VAS) score for infection damage**  (Measurement to 0.1cm allowed) | VAS measured  Yes*  No  Unknown | * At this time, overall disease damage **for infection** is:  ☐ ☐ ☐ ☐ ☐ ☐ ☐ ☐ ☐ ☐ ☐ ☐ ☐ ☐ ☐ ☐ ☐ ☐ ☐ ☐ ☐  0 0.5 1 1.5 2 2.5 3 3.5 4 4.5 5 5.5 6 6.5 7 7.5 8 8.5 9 9.5 10  Absent Maximum |
| **Malignancy** | Presence of malignancy | Yes  No  Unknown |  |
|  | **Visual Analogue Scale (VAS) score for malignancy (complications)**  (Measurement to 0.1cm allowed) | VAS measured  Yes*  No  Unknown | * At this time, overall disease damage **for malignancy** is:  ☐ ☐ ☐ ☐ ☐ ☐ ☐ ☐ ☐ ☐ ☐ ☐ ☐ ☐ ☐ ☐ ☐ ☐ ☐ ☐ ☐  0 0.5 1 1.5 2 2.5 3 3.5 4 4.5 5 5.5 6 6.5 7 7.5 8 8.5 9 9.5 10  Absent Maximum |
| **Any other damage** | Death | Yes *  No  Unknown | Date of death (year / month) /  Cause of death |
|  | **Visual Analogue Scale (VAS) score for any other disease damage**  (Measurement to 0.1cm allowed) | VAS measured  Yes*  No  Unknown | *Details of other damage ______________________________________________________  * At this time, overall disease damage **for any other disease damage** is:  ☐ ☐ ☐ ☐ ☐ ☐ ☐ ☐ ☐ ☐ ☐ ☐ ☐ ☐ ☐ ☐ ☐ ☐ ☐ ☐ ☐  0 0.5 1 1.5 2 2.5 3 3.5 4 4.5 5 5.5 6 6.5 7 7.5 8 8.5 9 9.5 10  Absent Maximum |
| **Global disease assessment - damage** | **Physician Visual Analogue Scale (VAS) score of Global disease damage**  (Measurement to 0.1cm allowed) | VAS measured  Yes*  No  Unknown | * At this time, overall **global** disease damage is:  ☐ ☐ ☐ ☐ ☐ ☐ ☐ ☐ ☐ ☐ ☐ ☐ ☐ ☐ ☐ ☐ ☐ ☐ ☐ ☐ ☐  0 0.5 1 1.5 2 2.5 3 3.5 4 4.5 5 5.5 6 6.5 7 7.5 8 8.5 9 9.5 10  Absent Maximum |

**GLOSSARY OF DEFINITIONS:**

This glossary of definitions has been designed to aid completion of variables within the Juvenile Dermatomyositis (JDM) Optimal Dataset and follows the same order and format of the dataset (Form A, Form B and Form C).

| **FORM A (FIRST / BASELINE VISIT): Glossary of definitions** | | |
| --- | --- | --- |
| **Category** | **Variable** | **Definition** |
| Demographics | Date of birth (year / month of birth +/- day of birth) | Year and month of birth, without day of birth if preferred. If date of birth not known by patient, an estimated date of birth should be given. |
|  | Sex of patient | Biological sex of patient - male or female. |
| Diagnostic factors | Date (year / month) of first symptom of myositis | Date (year and month) of first symptom of myositis - may include first symptom of weakness, cutaneous manifestation or associated feature (whichever presented first). |
|  | Date (year and month) of diagnosis of JDM | Date (year and month) of confirmed diagnosis of myositis. |
|  | Proximal muscle weakness at time of diagnosis of JDM | Symmetric muscle weakness affecting proximal muscles more than distal ones and sparing eye and facial muscles. Manual muscle testing or other objective strength testing should define muscle weakness. |
|  | Typical skin features of JDM (Gottron’s / Heliotrope) at time of diagnosis of JDM | Gottron’s Papules: Erythematous to violaceous papules (sometimes scaly) typically over the extensor surfaces of joints; may occur over the MCP / PIP joints, elbows, knees, malleoli and toes. Gottron’s sign: Erythematous to violaceous macules or patches (not palpable) over the extensor surfaces of joints. Heliotrope: Purple, lilac-coloured or erythematous patches over the eyelids or in a periorbital distribution, often associated with periorbital oedema. |
|  | MRI scan at diagnosis consistent with myositis (if an MRI scan was done) | MRI scan abnormal – consistent with myositis (STIR images showing muscle oedema). |
|  | Muscle biopsy consistent with myositis (if a muscle biopsy was done) | Muscle biopsy abnormal consistent with myositis - defined by validated muscle biopsy score [total score of 0-27] if possible; For muscle biopsy tool, refer to Varsani H et al, Annals Rheumatic Dis 2015; 74(1):204-10 |
|  | Myositis specific antibody (MSA) positive at diagnosis – if tested | Positive myositis specific antibody. If positive, please tick which one applies. |
|  | Myositis associated antibody (MAA) positive at diagnosis – if tested | Positive myositis associated antibody (more than one can be ticked where appropriate). |
| Treatments received prior to diagnosis of JDM | Has this patient received systemic glucocorticoids prior to diagnosis of JDM? | Systemic glucocorticoids (oral, intravenous or intramuscular) given prior to diagnosis of JDM, but within a time period that may alter the results of diagnostic investigations of JDM (in physician’s opinion) e.g. Patient taking at time of visit or within the preceding month. |
|  | Has this patient received synthetic or biologic disease modifying anti-rheumatic drug (DMARD) prior to diagnosis of JDM? | Synthetic (traditional DMARDS such as methotrexate) or biological DMARDS given prior to diagnosis of JDM, but within a time period that may alter the results of diagnostic investigations of JDM (in physician’s opinion) e.g. Patient taking at time of visit or within the preceding month. |

| **FORM B – FIRST AND EVERY VISIT [Since time of last data completion]: Glossary of definitions** | | |
| --- | --- | --- |
| **Category** | **Variable** | **Definition** |
| Growth | Height of patient | Height in centimetres on date of visit or most recently taken (monitor at regular intervals). |
|  | Weight of patient | Weight of patient in kilograms on date of visit or most recently taken (monitor at regular intervals). |
| Muscular involvement | Presence of symmetrical proximal muscle weakness | Symmetric muscle weakness affecting proximal muscles more than distal ones and sparing eye and facial muscles. Manual muscle testing or other objective strength testing should define muscle weakness. |
|  | CMAS score (carried out at regular time intervals to assess muscle strength / stamina; scored out of 52) | Childhood myositis assessment scale (CMAS) score: a validated measure of assessment of physical function in children. Maximum score = 52. Instructions can be found on the IMACS website; <https://www.niehs.nih.gov/research/resources/imacs/diseaseactivity/index.cfm> |
|  | MMT8 score (carried out at regular time intervals to assess muscle strength; scored out of 80) | MMT8 is a set of 8 designated muscles tested unilaterally (potential score 0 – 80), generally on right side (unless cannot be tested on right, then use left side). Calculation is always based on 8 scores. If less than 8 muscles tested the MMT8 cannot be calculated. Instructions can be found on the IMACS website: <https://www.niehs.nih.gov/research/resources/imacs/diseaseactivity/index.cfm> |
|  | Visual Analogue Scale (VAS) for muscle disease | Physician rated assessment of on-going symptoms and signs related to muscle disease only where 0 (left end of line) = no evidence of disease and right end of line = extreme or maximum amount of disease related to this section. Please record in centimetres (range = 0-10cm) with one digit after decimal point. |
| Skeletal involvement | Arthritis due to myositis | Inflammation, including swelling, warmth, tenderness, and / or redness of one or more joints detected by physical exam. |
|  | Contractures due to myositis | Limitation in range of movement of the joints in the absence of synovitis, excluding reducible deformities, avascular necrosis and deforming arthropathy. |
|  | VAS for skeletal disease | Physician rated assessment of on-going symptoms and signs related to skeletal disease only where 0 (left end of line) = no evidence of disease and right end of line = extreme or maximum amount of disease related to this section. Please record in centimetres (range = 0-10cm) with one digit after decimal point. |
| Cutaneous involvement | Presence of Gottron’s papules or Gottron’s sign | Gottron’s Papules: Erythematous to violaceous papules (sometimes scaly) typically over the extensor surfaces of joints; may occur over the MCP / PIP joints, elbows, knees, malleoli and toes. Gottron’s sign: Erythematous to violaceous macules or patches (not palpable) over the extensor surfaces of joints. |
|  | Presence of heliotrope rash | Purple, lilac-coloured or erythematous patches over the eyelids or in a periorbital distribution, often associated with periorbital oedema. |
|  | Presence of periungual capillary loop changes (where available to include measure of capillary density) | Periungual erythema with dilatation of periungual capillaries which may be accompanied by vessel dropout and which is visible by naked eye examination or by using additional magnification with an otoscope / ophthalmoscope / dermatoscope. Measure capillary density if possible. |
|  | Malar or facial erythema | Erythema over the face, which may be isolated malar erythema, but may include more extensive erythema including perioral, temporal, ear and frontal regions. |
|  | Linear extensor erythema | Erythema specifically located over the extensor tendon sheaths of the hands, forearms, feet and / or forelegs, which spares the skin over the extensor joint surfaces. |
|  | ‘V’ sign | Erythema over lower anterior neck and upper anterior chest. |
|  | Shawl sign | Erythema over the upper back, posterior neck and shoulders, sometimes extended to lateral arms. |
|  | Non-sun-exposed erythema | Erythema in areas not exposed to sun i.e. usually covered by clothing or in protected areas such as under the chin, flexural areas, palms, soles, trunk, groin. |
|  | Extensive cutaneous erythema, which may include erythroderma. | Generalised widespread confluent erythema involving both sun exposed and non-sun exposed skin with > 50% of body surface area involved [dermatologists define erythroderma as involving > 90% body surface area]. |
|  | Livedo reticularis | Net-like / lace-like mottling of the skin on the trunk or extremities due to a fixed peripheral vascular condition. |
|  | Cutaneous ulceration | Extensive injury to dermis, subcutaneous or deeper tissues due to dermatomyositis. |
|  | Mucus membrane lesions | Macules, dilated gingival capillaries, erosions / aphthae, or ulceration (injury to submucosa or deeper) involving oral, nasal, gingival, or genital region. |
|  | Mechanics hand’s | Lesions on the palmar or lateral aspects of the digits, which can include fissuring, cracking, hyperkeratosis, scaling, hyperpigmentation. |
|  | Cuticular overgrowth | Enlargement and overgrowth of the cuticle onto the nailbed. |
|  | Subcutaneous oedema | Swelling of skin and soft tissue, which may be localised – such as peri-orbital oedema (tissue swelling around the eyes) or may be widespread and generalised (anasarca). |
|  | Panniculitis | Inflammation of the subcutaneous fat causing painful erythematous or violaceous subcutaneous nodules or depressions. |
|  | Alopecia (non-scarring) | Hair loss – may be diffuse (non-scarring, non-erythematous widespread alopecia) or focal (patchy alopecia with scaling and erythema localised to areas of inflammation). |
|  | Calcinosis | Dystrophic calcium deposits, observed clinically or by imaging, which involves the skin, subcutaneous tissue, fascia, interfascial planes, muscle or across joints. |
|  | VAS for cutaneous disease | Physician rated assessment of on-going symptoms and signs related to skin disease only where 0 (left end of line) = no evidence of disease and right end of line = extreme or maximum amount of disease related to this section. Please record in centimetres (range = 0-10cm) with one digit after decimal point. |
| Features suggestive of myositis overlap | Does this patient have a myositis overlap condition? | If yes, options include overlap with (1) Scleroderma (2) Lupus (3) RNP positive Mixed Connective Tissue Disease (MCTD) or (4) Other – provide details. |
|  | Raynaud’s phenomenon | Discoloration of fingertips or other acral areas (two or three colours) to emotion or cold. |
|  | Sclerodactyly | Localised thickening and tightness of the skin of the fingers or toes, commonly associated with atrophy of the underlying soft tissues. |
| Gastro-intestinal involvement | Dysphagia due to myositis | Difficulty in swallowing or objective evidence of abnormal motility of the oesophagus. |
|  | Abdominal pain due to myositis | Abdominal pain due to myositis (exclude other causes of abdominal pain). |
|  | Gastrointestinal ulceration due to myositis | Gastrointestinal ulceration due to myositis (exclude peptic ulcer disease / other causes of ulceration). |
|  | VAS for gastrointestinal disease | Physician rated assessment of on-going symptoms and signs related to gastrointestinal disease only where 0 (left end of line) = no evidence of disease and right end of line = extreme or maximum amount of disease related to this section. Please record in centimetres (range = 0-10cm) with one digit after decimal point. |
| Pulmonary involvement | Pulmonary involvement / respiratory muscle weakness or interstitial lung disease due to myositis | Interstitial lung disease: Radiologic (chest x-ray or chest CT scan) documentation of inflammation or scarring (fibrosis) of the parenchyma of the lung. Abnormal pulmonary function tests attributable to inflammatory process or fibrosis. |
|  | Dysphonia due to myositis | Change in voice due to myositis. |
|  | VAS for pulmonary disease | Physician rated assessment of on-going symptoms and signs related to pulmonary disease only where 0 (left end of line) = no evidence of disease and right end of line = extreme or maximum amount of disease related to this section. Please record in centimetres (range = 0-10cm) with one digit after decimal point. |
| Cardiovascular involvement | Cardiovascular involvement due to myositis | Cardiac dysfunction including pericarditis, myocarditis or cardiomyopathy. Pericarditis: Inflammation of the pericardium defined clinically or by electrocardiogram (ECG) or echocardiogram; Myocarditis: Inflammation of the myocardium defined clinically or with echocardiographic or other objective evidence; Arrhythmia:  clinical or ECG evidence of irregular heartbeat, Sinus tachycardia: resting heart rate > greater than upper limit of age-appropriate normal value in a paediatric patient. |
|  | Blood pressure recording | Systolic and diastolic blood pressure measurement on the day of visit. |
|  | Blood pressure elevated suggesting hypertension | Hypertension defined by local population guidelines and age of patient. |
|  | VAS for cardiovascular disease | Physician rated assessment of on-going symptoms and signs related to cardiovascular disease only where 0 (left end of line) = no evidence of disease and right end of line = extreme or maximum amount of disease related to this section. Please record in centimetres (range = 0-10cm) with one digit after decimal point. |
| Constitutional features | Fever (>38^o^C) due to myositis | Ensure that other causes for fever are excluded. |
|  | Weight loss (>5%) due to myositis | Ensure that other causes of weight loss are excluded. |
|  | Fatigue due to myositis | A persistent feeling of tiredness / exhaustion / need to rest because of lack of energy or strength due to myositis. |
|  | VAS for constitutional features | Physician rated assessment of on-going symptoms and signs related to constitutional features only where 0 (left end of line) = no evidence of disease and right end of line = extreme or maximum amount of disease related to this section. Please record in centimetres (range = 0-10cm) with one digit after decimal point. |
| Global disease assessment by clinician | Physician VAS of global disease | Physician VAS – global activity (overall): Disease activity defined as potentially reversible pathology or physiology resulting from myositis. Clinical findings known or suspected to be due to another disease should not be considered. Rate your global activity assessment judged by all the information available to you today including the patient’s appearance, history, physical examination and laboratory tests. Please record in centimetres (range = 0-10cm) with one digit after decimal point. |
|  | Physician VAS of extra-muscular disease | Physician VAS – extra-muscular global activity: Encompasses an overall evaluation for the disease activity in all the extra-muscular organ systems EXCLUDING muscle disease activity. Please record in centimetres (range = 0-10cm) with one digit after decimal point. |
| Global disease assessment by patient / parent | Patient / parent VAS for global disease | Parental / patient VAS: The global evaluation by the patient, or by the parent if the patient is a minor, of the patient's overall disease activity at the time of assessment using a 10-cm visual analogue scale, where 0 (left end of line) = no evidence of disease and right end of line = extreme or maximum amount of disease. Please record in centimetres (range = 0-10cm) with one digit after decimal point. |
|  | Patient / parent VAS for pain | Parental / patient VAS: The global evaluation by the patient, or by the parent if the patient is a minor, of the patient's overall pain at the time of assessment using a 10-cm visual analogue scale, where 0 (left end of line) = no pain and right end of line = extreme or maximum amount of pain. Please record in centimetres (range = 0-10cm) with one digit after decimal point. |
| Patient / parent reported outcome measure (PROM) | Use of an age-appropriate patient / parent reported measure of function | A measure of activity / function appropriate for local population – e.g. Childhood Health Assessment Questionnaire [CHAQ] / Health Assessment Questionnaire [HAQ]. |
|  | Use of an age-appropriate patient / parent reported measure of quality of life | A health-related quality of life / measure of level of participation appropriate for local population e.g. Child Health Questionnaire [CHQ-50]. |
| Investigations | Elevation of any muscle enzyme (including CPK, LDH, aldolase, AST / SGOT, ALT / SGPT) above normal range | For all enzymes: Enzyme level above the upper range of normal according to hospital’s normal reference range. Muscle enzymes include: (1) Creatine phosphokinase (CPK), (2) Lactate dehydrogenase (LDH), (3) Aldolase (not universally available), (4) Aspartate Aminotransferase (AST) / Serum Glutamic Oxaloacetic Transaminase (SGOT) – different names for the same enzyme, (5) Alanine transaminase (ALT) / Serum glutamic-pyruvic transaminase (SGPT) – different names for the same enzyme. |
| Specimens available | Has this patient had specimens taken that may be available for specific research projects? This may include DNA, serum, biomarkers, biopsy tissue or other material. | This is simply to record the possible availability of specimens for future research studies and does not commit the physician / patient / parent to these being used.  If the answer to this question is yes, please tick boxes as appropriate to show:   1. Has this patient had DNA taken that may be available for specific research projects? 2. Has this patient had serum taken that may be available for specific research projects? 3. Has this patient had any other samples / tissue taken that could be used for future studies? |
| Treatment | Is the patient on treatment (now or since last visit)? | For all drugs: Patient taking at time of visit (or recently taken since time of last data entry). This includes corticosteroids (oral, intravenous or intramuscular), synthetic DMARDS (e.g. Methotrexate, ciclosporin A, mycophenolate mofetil etc) or biologic DMARDS (e.g. rituximab / anti-TNF therapies) or other immunotherapy (e.g. Intravenous immunoglobulin). For each medication that the patient is on state dose, route of administration and frequency of administration. |
|  | Is the patient doing a regular exercise routine prescribed by a healthcare professional aimed at improving / maintaining:   1. Range of movement 2. Muscle strength | At time of visit or since last data entry. |

| **FORM C: FIRST & ANNUAL VISIT: Glossary of definitions**  **NB: Items need to be present for > 6 months, not responding to immunosuppressive therapy and not part of active inflammation to qualify as damage** | | |
| --- | --- | --- |
| **Category** | **Variable** | **Definition** |
| Muscle damage | Muscle atrophy (clinical) | Decreased muscle mass assessed by clinical examination. |
|  | Muscle weakness not attributable to active muscle disease | Weakness present for at least 6 months, demonstrated on clinical examination, not thought to be due to active muscle inflammation based on assessments of clinical and laboratory measures (such as muscle enzymes, repeat MRI or repeat muscle biopsies). |
|  | Muscle dysfunction: decrease in aerobic exercise capacity | Decrease in aerobic exercise tolerance by clinical history or assessed by aerobic exercise testing, due to muscle damage and not attributable to cardiac, pulmonary or psychological or other factors. |
|  | VAS for muscle damage | Physician rated assessment of disease damage related to muscle disease only, where 0 (left end of line) = no evidence of damage and right end of line = extreme or maximum amount of damage related to this section. Please record in centimetres (range = 0-10cm) with one digit after decimal point. |
| Skeletal damage | Joint contractures (due to myositis) | Fixed limitation in range of movement of the joints in the absence of synovitis, excluding reducible deformities, avascular necrosis and deforming arthropathy. |
|  | Osteoporosis with fracture or vertebral collapse (excluding avascular necrosis) | Demonstrated by any imaging technique. |
|  | Avascular necrosis | Demonstrated by any imaging technique. |
|  | Deforming arthropathy | Including reducible deformities, excluding avascular necrosis and contractures. |
|  | VAS for skeletal damage | Physician rated assessment of disease damage related to skeletal disease only, where 0 (left end of line) = no evidence of damage and right end of line = extreme or maximum amount of damage related to this section. Please record in centimetres (range = 0-10cm) with one digit after decimal point. |
| Cutaneous damage | Calcinosis (persistent) | Dystrophic calcification observed clinically or radiologically in the skin, subcutaneous tissue, fascia or muscle (includes superficial plaques / nodules, tumoral, planar, exoskeleton / calcinosis universalis) present for > 6 months, not responding to immunosuppressive therapy and not part of active inflammation. |
|  | Alopecia (scarring) | Permanent hair loss leaving shiny skin with lack of hair follicles. |
|  | Cutaneous scarring or atrophy | Depressed scar or cutaneous atrophy: End stage of lesions due to vascular occlusion or vascular insufficiency manifested as depressions or skin thinning. |
|  | Poikiloderma | Poikiloderma: A fine speckled pattern of hyperpigmented and hypopigmented macules interspersed with fine telangiectasia and cutaneous atrophy (requires all 3 features). |
|  | Lipoatrophy / lipodystrophy | Loss of subcutaneous fat – localised or widely distributed, observed clinically or radiologically. |
|  | VAS for cutaneous damage | Physician rated assessment of damage related to skin disease only, where 0 (left end of line) = no evidence of damage and right end of line = extreme or maximum amount of damage related to this section. Please record in centimetres (range = 0-10cm) with one digit after decimal point. |
| Gastrointestinal damage | Dysphagia (persistent) | Persistent (> 6 months) difficulty swallowing by history or persistent changes documented by radiography or other objective measures. |
|  | Gastrointestinal dysmotility, constipation, diarrhoea or abdominal pain (persistent) | Persistent gastrointestinal symptoms (dysmotility / constipation / diarrhoea / abdominal pain) due to myositis. |
|  | Infarction or resection of bowel or other gastrointestinal organs | By history |
|  | VAS for gastrointestinal damage | Physician rated assessment of damage related to gastrointestinal disease only, where 0 (left end of line) = no evidence of damage and right end of line = extreme or maximum amount of damage related to this section. Please record in centimetres (range = 0-10cm) with one digit after decimal point. |
| Pulmonary damage | Dysphonia (persistent) | Persistent (> 6 months) alteration in voice quality, resonance, articulation or voice rate from normal. |
|  | Impaired lung function due to respiratory muscle damage | Shortness of breath not thought to be due to active muscle inflammation or intrinsic pulmonary disease. |
|  | Pulmonary fibrosis | Shortness of breath or rales (abnormal rattling sound) on physical examination for at least 6 months, with previously documented abnormal chest radiograph, CT or biopsy evidence of interstitial lung disease / pulmonary fibrosis. |
|  | Pulmonary hypertension | Right ventricular prominence or loud P2 or by direct measurement of pulmonary pressures (>10% above upper limit of normal). |
|  | VAS for pulmonary damage | Physician rated assessment of damage related to pulmonary disease only, where 0 (left end of line) = no evidence of damage and right end of line = extreme or maximum amount of damage related to this section. Please record in centimetres (range = 0-10cm) with one digit after decimal point. |
| Cardiovascular damage | Hypertension requiring treatment for > 6 months | Blood pressure > 95% of upper limits of age and gender, requiring treatment for > 6 months. |
|  | Ventricular dysfunction or cardiomyopathy | Ventricular dysfunction documented clinically or by echocardiography. |
|  | Assessed in adults (> 18 years of age) only: Angina or coronary artery bypass | Episodes of angina present for a period of at least 6 months. Can be completed in patients < 18 years of age where applicable. |
|  | Assessed in adults (> 18 years of age) only: Myocardial infarction | Documented by electrocardiogram and enzymes. Can be completed in patients < 18 years of age where applicable. |
|  | VAS for cardiovascular damage | Physician rated assessment of damage related to cardiovascular disease only, where 0 (left end of line) = no evidence of damage and right end of line = extreme or maximum amount of damage related to this section. Please record in centimetres (range = 0-10cm) with one digit after decimal point. |
| Peripheral vascular damage | Tissue or pulp loss | Tissue loss such as pulp space loss or loss less than entire digit. |
|  | Digit loss or limb loss or resection | Loss of an entire digit or limb. |
|  | Venous or arterial thrombosis with swelling, ulceration or venous stasis | Thrombosis confirmed using imaging techniques. |
|  | Assessed in adults (>18 years of age): Claudication | By history. Can be completed in patients < 18 years of age where applicable. |
|  | VAS for peripheral vascular damage | Physician rated assessment of damage related to peripheral vascular disease only, where 0 (left end of line) = no evidence of damage and right end of line = extreme or maximum amount of damage related to this section. Please record in centimetres (range = 0-10cm) with one digit after decimal point. |
| Pubertal Status | Pubertal status of patient – defined by Tanner score (self-assessment forms can be used) | Using a self-reported pubertal assessment [Rasmussen AR et al, Pediatrics 2015;135:86-93] or physician assessment. The patient or physician defines the Tanner score at the time of visit. |
| Endocrine damage | Growth failure | Two of the following 3 features: Two of the following 3 features: (1) Less than 3rd percentile height for age (2) Growth velocity over 6 months less than 3rd percentile for height (3) Crossing at least 2 centiles on growth chart. |
|  | Delay in development of secondary sexual characteristics | Greater than 2 standard deviations beyond mean for age in Tanner staging. |
|  | Hirsutism or hypertrichosis | Hirsutism: Excessive terminal hair growth (in females / pre-pubescent boys) in an adult male distribution. Hypertrichosis: generalised increase in body hair. |
|  | Irregular menses | Missing more than one ovulatory menstrual cycle in one year, assessed at least 3 years after menarche. |
|  | Primary or secondary amenorrhea | Primary amenorrhoea: absence of any menstruation within 4.5 years of reaching puberty. Secondary amenorrhoea: cessation of menstrual periods before menopause, after initially menstruating. |
|  | Diabetes mellitus | Fasting glucose > 140mg/dl (7.8mmol/L) or 2-hour glucose in oral glucose tolerance test > 200mg/dl (11.1mmol/L) |
|  | In adults (> 18 years of age): Infertility – male or female | No pregnancy after 1 year attempting to conceive via regular sexual intercourse. Can be completed in patients < 18 years of age where applicable. |
|  | In adults (>18 years of age): Sexual dysfunction | Patient dissatisfaction with sexual function (female or male). Can be completed in patients < 18 years of age where applicable. |
|  | VAS for endocrine damage | Physician rated assessment of damage related to endocrine disease only, where 0 (left end of line) = no evidence of damage and right end of line = extreme or maximum amount of damage related to this section. Please record in centimetres (range = 0-10cm) with one digit after decimal point. |
| Ocular damage | Cataract resulting in visual loss | Lens opacity in either eye, ever, whether primary or secondary to steroid therapy, documented by ophthalmoscopy, and resulting in visual loss. |
|  | Visual loss, other, not secondary to cataract | Legal blindness with vision less than or equal to 20/200. |
|  | VAS for ocular damage | Physician rated assessment of damage related to ocular disease only, where 0 (left end of line) = no evidence of damage and right end of line = extreme or maximum amount of damage related to this section. Please record in centimetres (range = 0-10cm) with one digit after decimal point. |
| Infection | Chronic infection | Infection (presumptive diagnosis with or without positive culture) requiring > 6 months of antimicrobial treatment or persisting with or without clinical symptoms > 6 months and associated with disability (absence from school, work, day care). |
|  | Multiple infections | >3 infections (at same or different sites, with same or different organisms) requiring antimicrobial treatment or disability (absence from school, work, day care) over a 6-month period. |
|  | VAS for infection | Physician rated assessment of damage related to infective complications only, where 0 (left end of line) = no evidence of damage and right end of line = extreme or maximum amount of damage related to this section (maximum level guidance = infection resulting in septic shock or life-threatening complications). Please record in centimetres (range = 0-10cm) with one digit after decimal point. |
| Malignancy | Presence of malignancy | Regardless of whether considered to be myositis associated, documented by pathology, excluding dysplasias. |
|  | VAS for malignancy (complications) | Physician rated assessment of damage related to malignancy complications only, where 0 (left end of line) = no evidence of damage and right end of line = extreme or maximum amount of damage related to this section (maximum level guidance = malignancy resulting in ICU care or life-threatening complications). Please record in centimetres (range = 0-10cm) with one digit after decimal point. |
| Other damage | Death | Note date and cause of death. |
|  | VAS for other damage (provide details of other damage) | Physician rated assessment of damage related to features of myositis damage not listed but felt to be of importance to the physician or clinician, where 0 (left end of line) = no evidence of damage and right end of line = extreme or maximum amount of damage related to this section (maximum level guidance = resulting in ICU care or life-threatening complications). Please record in centimetres (range = 0-10cm) with one digit after decimal point. |
| Global damage assessment | Physician VAS of Global damage | Physician VAS – global damage (overall): Disease damage defined as persistent changes in anatomy, pathology or function, which are present for at least 6 months. The global damage score is the physician’s expert judgment of the totality of damage in all systems. Please record in centimetres (range = 0-10cm) with one digit after decimal point. |

| **Manual Muscle Testing abbreviated score [MMT8] scoring sheet:** | | |  |
| --- | --- | --- | --- |
| **Muscle group** | **Starting (test) position** | **Details for testing**  (Test right side only) | **Score**  [0-10] |
| Shoulder abduction (Deltoid) | With patient sitting, shoulder abducted at 90 degrees; patient to maintain arm in abduction against gravity. | Place pressure against the dorsal surface of the forearm with the elbow extended (or against distal end of humerus if elbow flexed). |  |
| Elbow flexion (Biceps) | With patient sitting, elbow flexed at 45 degrees with forearm in supination. | Add resistance over the flexor surface of the forearm, proximal to the wrist. |  |
| Wrist extension | With patient sitting, hand pronated and wrist extended 60 degrees with fingers flexed. | Support the forearm in its neutral position and apply resistance to the back of the hand. |  |
| Knee extension | With patient sitting over the edge of a bed / examination couch (trunk perpendicular to floor), knee extended but not locked (allow 5 degrees flexion). | Place resistance above ankle, keeping other hand on knee to ensure the knee does not lock. If not possible due to tight hamstrings, patient can be allowed to lean back onto elbows with trunk extended. |  |
| Ankle dorsiflexion | With patient sitting and knee flexed to 90 degrees, ankle dorsi-flexed to 5 degrees. | Add resistance to the dorsal aspect of the foot. |  |
| Neck flexion | Patient lying completely supine with arms at their side, lifting neck up, flexing to about 45 degrees. | Place resistance to the forehead. |  |
| Hip abduction (Gluteus medius) | Patient lying on their side with slight extension at the pelvis and forward tilt at the trunk, abduct the hip, keeping the knee of the test leg straight (supporting leg can be flexed for stability). | Keeping top leg slightly extended backwards, pelvis slightly rolled forward, abduct top leg, stabilise the pelvis with one hand and add resistance proximal to the knee. |  |
| Hip extension (Gluteus maximus) | Patient lying prone with the pelvis flat and knee flexed to 90 degrees. | Keeping knee flexed, lift the upper leg and knee up off the bed to about 15 degrees, place resistance on the back of the leg close to the knee. |  |
| **TOTAL MMT8 SCORE / 80** | | |  |
| **MMT8 Scoring Guidance:**  0 = no movement / no contraction felt in the muscle.  1 = moves through partial range of movement in horizontal plane.  2 = moves through complete range of movement in horizontal plane.  3 = moves to completion of range of movement against resistance or moves to completion of range and holds against pressure or moves through partial range of movement in an anti-gravity position.  4 = gradual release from test position in an anti-gravity position.  5 = holds against test position (no added pressure).  6 = Holds test position against slight pressure.  7= holds test position under slight to moderate pressure.  8 = holds test position under moderate pressure.  9 = holds test position under moderate to strong pressure.  10 = holds test position against strong pressure. | | |  |
| Further details available on IMACS website, including gravity eliminated positions for weaker patients: <https://www.niehs.nih.gov/research/resources/assets/docs/mmt8_grading_and_testing_procedures_for_the_abbreviated_8_muscle_groups_508.pdf> | | |  |

| **Childhood Myositis Assessment Scale [CMAS] scoring sheet** | | | |
| --- | --- | --- | --- |
|  | **Test** | **Details for scoring** | **Item score:** |
| 1 | Head elevation (neck flexion) | 0 = Unable  1 = 1-9 seconds  2 = 10-29 seconds  3 = 30-59 seconds  4 = 60-119 seconds  5 = ≥ 2 minutes | Item score ____ /5  Number of seconds_______ |
| 2 | Leg raise / touch object | 0 = Unable to lift leg of the table.  1 = Able to clear table, but cannot touch object (examiner’s hand).  2 = Able to lift leg high enough to touch object (examiner’s hand). | Item score ____ /2 |
| 3 | Straight leg lift / duration | 0 = Unable  1 = 1-9 seconds  2 = 10-29 seconds  3 = 30-59 seconds  4 = 60-119 seconds  5 = ≥ 2 minutes | Item score ____ /5 |
| 4 | Supine to prone | 0 = Unable. Has difficulty even turning onto side; able to pull right arm under torso only slightly or not at all.  1 = Turns onto side fairly easily, but cannot fully free right arm and is unable to fully assume a prone position.  2 = Easily turns onto side, has some difficulty freeing arm, but fully frees arm and assumes a prone position.  3 = Easily turns over, fully frees right arm with no difficulty. | Item score ____ /3 |
| 5 | Sit ups  For each type of sit up enter 0 (unable) / 1 (able), then enter total score (maximum =6) | - Hands on thighs with counterbalance - Hands across chest with counterbalance ___ - Hands behind head with counterbalance ___ - Hands on thighs without counterbalance ___ - Hands across chest without counterbalance ___ - Hands behind head without counterbalance ___ | Item score ____ /6 |
| 6 | Supine to sit | 0 = Unable by self  1 = Much difficulty. Very slow, struggles greatly, barely makes it, almost unable.  2 = Some difficulty. Able but is somewhat slow, struggles some.  3 = No difficulty. | Item score ____ /3 |
| 7 | Arm raise / straighten | 0 = Cannot raise wrists up to level of the acromioclavicular joint.  1 = Can raise wrists at least up to the level of the acromioclavicular joint but not above top of head.  2 = Can raise wrists above top of head but cannot raise arms straight above head so that elbows are in fully extension.  3 = Can raise arms straight above head so that elbows are in fully extension. | Item score ____ /3 |
| 8 | Arm raise / duration | 0 = Unable  1 = 1-9 seconds  2 = 10-29 seconds  3 = 30-59 seconds  4 = ≥ 60 seconds | Item score ____ /4 |
| 9 | Floor sit:  Going from a standing position to a sitting position on the floor | 0 = Unable. Afraid to even try, even if allowed to use a chair for support. Child fears that he / she will collapse, fall into a sit, or harm self.  1 = Much difficulty. Able, but needs to hold onto a chair for support during decent. Unable or unwilling to try if not allowed to use a chair for support.  2 = Some difficulty. Can go from stand to sit without using a chair for support, but has at least some difficulty during decent. May need Gower’s. Descends somewhat slowly and / or apprehensively; may not have full control of balance as manoeuvres into sit.  3 = No difficulty. Requires no compensatory manoeuvring. | Item score ____ /3 |
| 10 | All fours manoeuvre | 0 = Unable to go from prone to an all fours position.  1 = Barely able to assume and maintain all fours position. Unable to raise head to look straight ahead.  2 = Can maintain all fours position with back straight and head raised (so as to look straight ahead) but cannot creep (crawl) forward.  3 = Can maintain all fours, look straight ahead and creep (crawl) forward.  4 = Maintains balance while lifting and extending one leg. | Item score ____ /4 |
| 11 | Floor rise:  Going from a kneeling position on the floor to a standing position. | 0 = Unable, even if allowed to place hands on side of chair.  1 = Much difficulty. Able but needs to place hands on sides of chair.  2 = Moderate difficulty. Able but needs to place one or both hands on thighs / knees or floor (unable without using hands).  3 = Mild difficulty. Does not need to place hands on knees / thighs or floor but has at least some difficulty during ascent.  4 = No difficulty. | Item score ____ /4 |
| 12 | Chair rise | 0 = Unable to rise up from chair, even if allowed to place hands on sides of chair seat.  1 = Much difficulty. Able, but needs to place hands on side of seat. Unable if not allowed to place hands on sides of seat.  2 = Moderate difficulty. Able, but needs to place hands on knees / thighs. Does not need to place hands on side of seat.  3 = Mild difficulty. Does not need to place hands on seat, knees or thigh, but at least some difficulty during ascent.  4 = No difficulty. | Item score ____ /4 |
| 13 | Stool step | 0 = Unable.  1 = Much difficulty. Able but needs to place one hand on exam table (or examiner’s hand).  2 = Some difficulty. Able, does not need to use exam table for support but does need to use hand on knee / thigh.  3 = Able. Does not need to use exam table or hand on knee / thigh. | Item score ____ /3 |
| 14 | Pick-up: | 0 = Unable to bend over and pick up a pencil from the floor.  1 = Much difficulty. Able, but relies heavily on support gained by placing hands on knees / thighs.  2 = Some difficulty. Has some difficulty (but not ‘much difficulty’). Needs at least minimally and briefly place hand(s) on knees / thighs for support. Is somewhat slow.  3 = No difficulty. No compensatory manoeuvre necessary. | Item score ____ /3 |
| **TOTAL CMAS SCORE / 52** | | |  |
| Instructions for completing the CMAS score can be found on the IMACS website: <https://www.niehs.nih.gov/research/resources/assets/docs/cmas_instructions_508.pdf> | | |  |
