# Supplementary figures and images for: Development of a consensus core dataset in juvenile dermatomyositis for clinical use to inform research
Source: Ann Rheum Dis. 2017 Oct 30;77(2):241–50. doi: 10.1136/annrheumdis-2017-212141 (PMC5816738; doi:10.1136/annrheumdis-2017-212141)

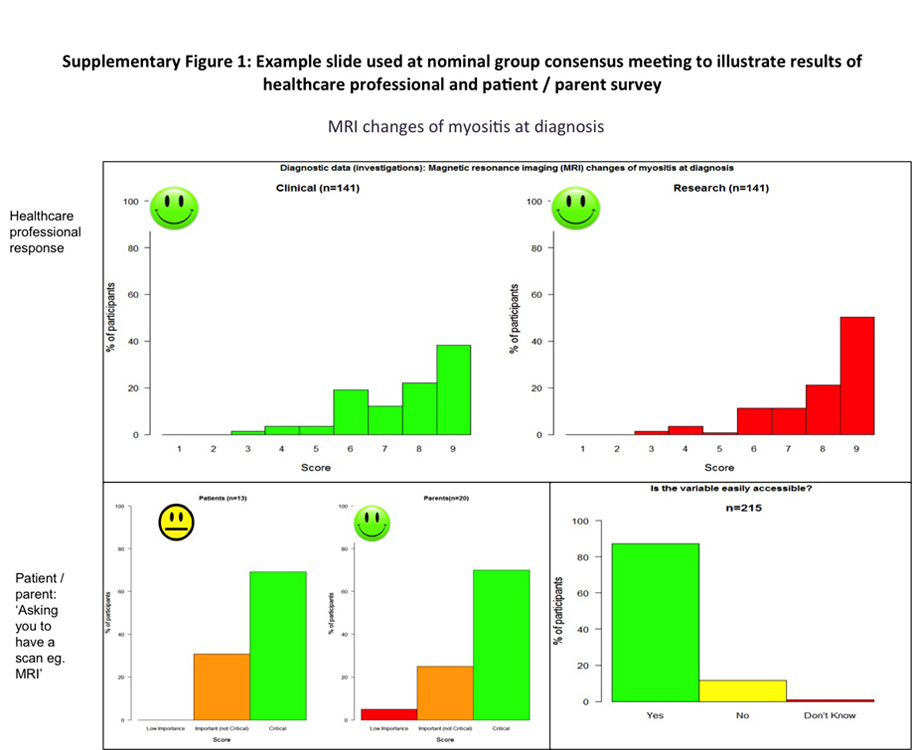

Supplement: Supplementary file 2 [file annrheumdis-2017-212141supp002.jpg]
